# Tuning photoluminescence in Gd_2_O_3_ via lattice engineering for advanced barcode and LEDs applications

**DOI:** 10.1038/s41598-025-21434-3

**Published:** 2025-10-28

**Authors:** Praful P. Khode, Merciana N. Sylvester, Nikolay G. Naumov, Anil M. Pethe, S. J. Dhoble

**Affiliations:** 1 Department of Physics, R.T.M. Nagpur University, Nagpur, 440033 India; 2 Institute of Inorganic Chemistry, Siberian Branch of the Russian Academy of Science, Novosibirsk, Russia 630090; 3 Datta Meghe College of Pharmacy, Datta Meghe Institute of Higher Education and Research, Deemed to be University, Sawangi (Meghe), Wardha, India

**Keywords:** LEDs phosphor, Lanthanides, Barcode, Color tunability, Photoluminescence, Chemistry, Materials science, Nanoscience and technology, Optics and photonics

## Abstract

The utilization of luminescent phosphors in barcode technology can enhance the security feature. Using NaOH as a reducing agent, we have prepared Gd_2_O_3_:Eu^3+^/Er^3+^ for luminescent phosphor. The cubic structure of Gd_2_O_3_ phosphor is confirmed by the structural analysis conducted using XRD. Dopant Eu and Er induce lattice contraction and expansion in the host lattice, respectively. Based on SEM analysis, porous and irregular particles with an average size of 401 nm were observed. In Tem analysis of prepared phosphor have some particle are in spherical in shape. The formation of Gd_2_O_3_ was confirmed by Gd-O band stretching as shown in the FTIR analysis. The emission in the red and green regions is emitted by Gd_2_O_3_:Eu^3+^ and Gd_2_O_3_:Er^3+^, respectively. The emission peaks of Gd_2_O_3_:Eu^3+^/Er^3+^ are observed at 524 nm, 539 nm, 549 nm, 563 nm, 593 nm, 612 nm, and 629 nm when it is triggered by 363 nm, with a maximum energy transfer efficiency of 95.93%. Enhancing the security of barcodes can be achieved through the use of color-tunable Gd_2_O_3_:Eu^3+^/Er^3+^ phosphor. Gd_2_O_3_:Er^3+^ (1.5 mol%) is a prominent candidate for LED bulbs due to its CCT (5765 K).

**Supplementary Information:**

The online version contains supplementary material available at 10.1038/s41598-025-21434-3.

## Introduction

In recent years, the security and authenticity of barcodes have become a significant concern, prompting researchers to develop advanced systems aimed at enhancing barcode protection and preventing counterfeiting^1,2^. A common technology that sends a bundle of data about objects and packages is barcodes. Contactless payment, device pairing, product information, URL incorporation into barcodes, and mobile advertising are some of the more important applications for 2D barcode technology these days. Supply chain management (SCM) relies heavily on barcodes because of their dependability and low cost. An additional layer of security can be added to a 2D barcode by adding a color-tunable phosphor. This kind of phosphor-enabled barcode is helpful in a lot of high-security applications, like pharmaceuticals, supply chains, banking, defense, and secure IDs (in places that are extremely secure, like government agencies, border control, military access, and banks) to stop counterfeiting, guarantee authenticity, and safeguard private information^3–5^.

Nowadays, tuning the physical and chemical properties of metal oxide attracts plenty of attention from both a fundamental and technological perspective. Changing the optical characteristics of the host module has significant applications in barcodes, display devices, etc.^6^.

Luminescent lanthanides have had wide applications over the last decade in various fields such as biosciences, electroluminescent devices, photodynamic therapy, optoelectronics, luminescent probes in biological systems, biomedicine, and solar energy conversion devices^6–8^. Photoluminescence (PL) property of lanthanide is unique due to it giving line-like emission with long decay time (microsecond to millisecond). Transition in lanthanide ions such as Gd, Er, Dy, Eu, Sm, and Yb, etc. is due to f-f transition for up-conversion and f-d down-conversion processes. Gd_2_O_3_ is a paramagnetic material with excellent chemical and optical properties. Gd_2_O_3_ has high band–gap energy (5.6 eV), low photon energy about 600 cm^−1^, good chemical and thermal stability, and high refractive index. It has a high melting point of about 2400 °C, high crystallographic stability of up to 2325 °C, high thermal conductivity, and high mechanical strength that makes it a more appropriate choice for applications such as display applications, WLEDs, luminescent paints, sensors, optoelectronic devices, and other luminescent application. Gd_2_O_3_ is also used as a host material for coating solar cells to enhance the efficiency of solar cells^9^ . PL emission of the host depends on the activator such as Eu is commonly used for red emission due to *d–f* transitions while Tb (terbium) is used for green emission with *d–f* transitions^10^, while Sm (samarium) emits orange-red emission due to available *g–h* transitions^11^ and, Dy (dysprosium) is emits blue and yellow emission to form white due to *f–h* transitions^12^. All lanthanide activators give sharp emissions in their respective emission while some transition metals such as Bi give blue board emissions while Mn gives red and blue emission depending on oxidation state of Mn in the host lattice^13^. A single activator is typically associated with one or more specific color emissions. So achieving the color tuning in a single host lattice with a single activator is not possible therefore we co-doped the two or more activator ions to achieve the color tunable phosphor in a single phosphor^14–17^.

Vishnu V. Jaiswal et. al. reported the Ca_0.87_Al_2_O_4_:0.01Eu^2+^,0.02Nd^3+^,0.1Si^4+^ phosphor for the barcode application prepared by solid-state method and its photoluminescence property is studied. In his study, they observed different shades of the blue color to secure the barcode^18^. Ranjoy Wangkhem et. al. reported Gd_2_O_3_:Eu^3+^ phosphor prepared by hydrothermal method. In his study, he includes the photoluminescence property of Gd_2_O_3_:Eu^3+^^19^. D. Šević et. al. reported the Gd_2_O_3_:Er^3+^ phosphor using solution combustion method. In his study, they studied the up conversion photoluminescence property for temperature sensing^20^.

In this study, we synthesized the Gd_2_O_3_:Eu^3+^, Gd_2_O_3_:Er^3+^, and Gd_2_O_3_:Er^3+^/Eu^3+^ samples by using the co-precipitation method by using NaOH as a reducing agent. For structural analysis of the prepared sample, we obtained x-ray diffraction (XRD), morphological is studied by scanning electron microscopy (SEM) and Transmission Electron Microscope (TEM), starching between the bonds studied by using Fourier transform infrared Spectroscopy (FT-IR), and photoluminescence (PL) property is studied. So prepared phosphor can be prominent candidate for red LEDs, green LEDs and barcode application.

## Characterization techniques and instrument details

XRD pattern of the above sample is carried out by using a Rigaku Miniflex D 600 X-ray diffractometer enabled with Cu Kα radiation (λ = 0.154056 nm), operating at 40 kV and 15 mA. XRD pattern is recorded from over a 2θ range of 10–80° with a step size of 0.04°. SEM images of prepared phosphor was taken by using a ZEISS-EVO-18 scanning electron microscope. TEM images were obtained by using 200 kV FEG Transmission Electron Microscope (TEM) with Schottky Field Emission Gun with energy resolution ≤ 0.8 eV. The FTIR of synthesized phosphor was carried out using a brucker alpha FT-IR spectrometer. The photoluminescence emission and excitation spectra were obtained using shimadzu RF5301PC spectroflurometerphotometer with excitation source of xenon lamp (150 W). The CIE co-ordinate result were calculated using OSRAM SYLVANIA colour calculator.

## Synthesis

**Fig. 1:**
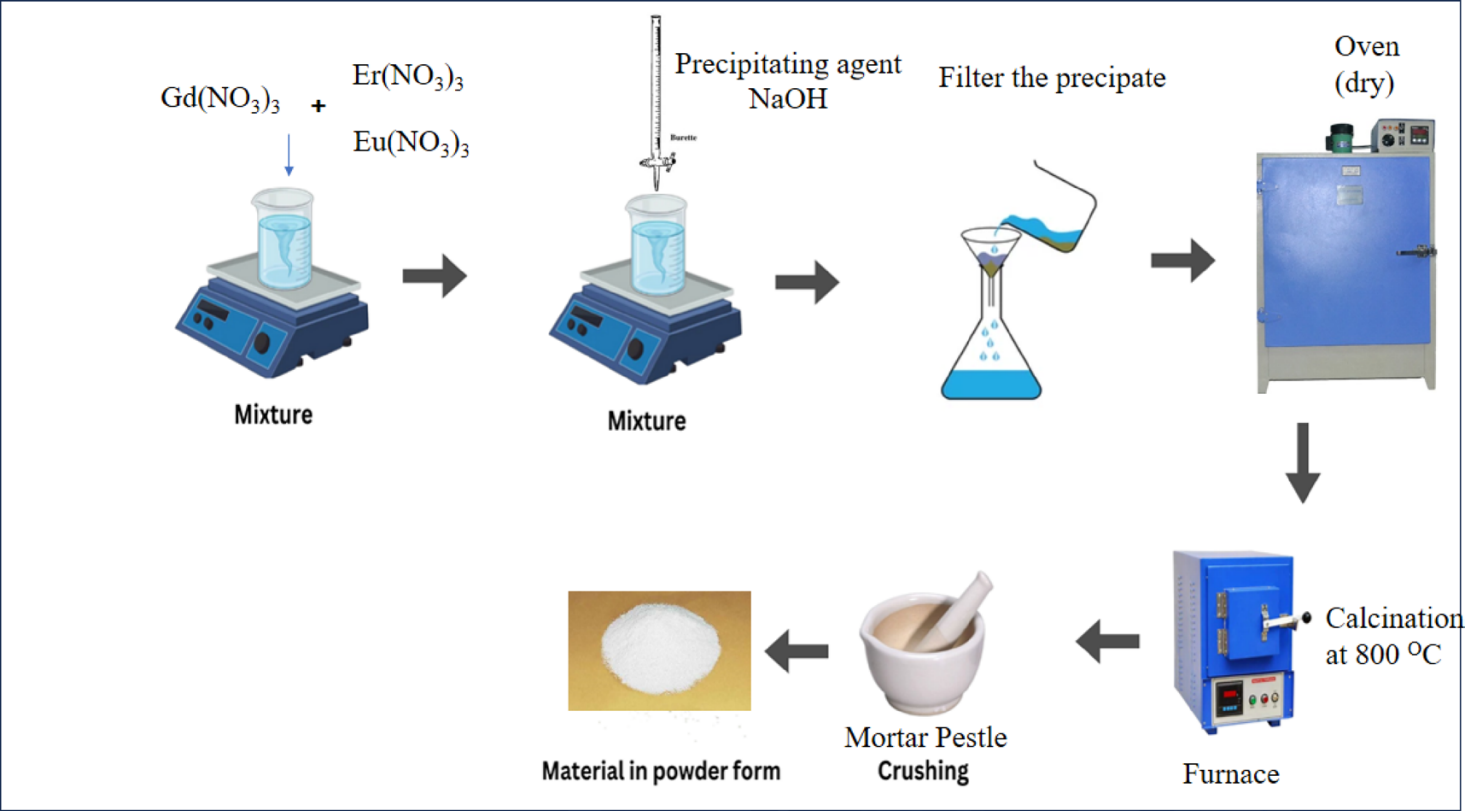
Systematic diagram representation of co-precipitation method.

Gd_2_O_3_, Gd_2_O_3_:Eu^3+^, Gd_2_O_3_:Er^3+^, and Gd_2_O_3_:Er^3+^/Eu^3+^ phosphors were prepared by co-precipitation method. Gadolinium (III) Nitrate Hexahydrate (Gd(NO_3_)_3_*6H_2_O)ultrapure, 99.99%, Europium (III) Oxide extra-pure, 99.9% (Eu_2_O_3_) and Erbium (III) Oxide ultrapure, 99.99% (Er_2_O_3_) were starting precursor. After weighing, Eu₂O₃ and Er₂O₃ were added to a test tube containing concentrated nitric acid (HNO₃, 69% purity) to convert the insoluble oxides into their soluble nitrates, and the resulting solution was then placed in an oven at 80 °C. The Gd(NO_3_)_3_*6H_2_O was added in a beaker with double distilled water and placed on a magnetic starrier with a constant rate of 250 rpm. A solution containing Er and Eu ions was added to the Gd solution. 0.1 M NaOH solution was added slowly in the solution drop by drop at a rate of 10 drops per minute. solution of precipitating agent (NaOH) was added to reach pH of resultant solution to 8. After that precipitate was goes through the process of filtration and washed with double distilled water 2 times. Precipitate was dried in an oven for 8 h at 80 °C. Dried sample was crushed for 5 min and placed in an oven for calcination at 800 °C for 8 h. Sample was again crushed for 2 min and sent for further characterization. The schematic representation of synthesis is shown in Fig. 1.

## Result and discussion

### XRD and crystal structure analysis

**Fig. 2:**
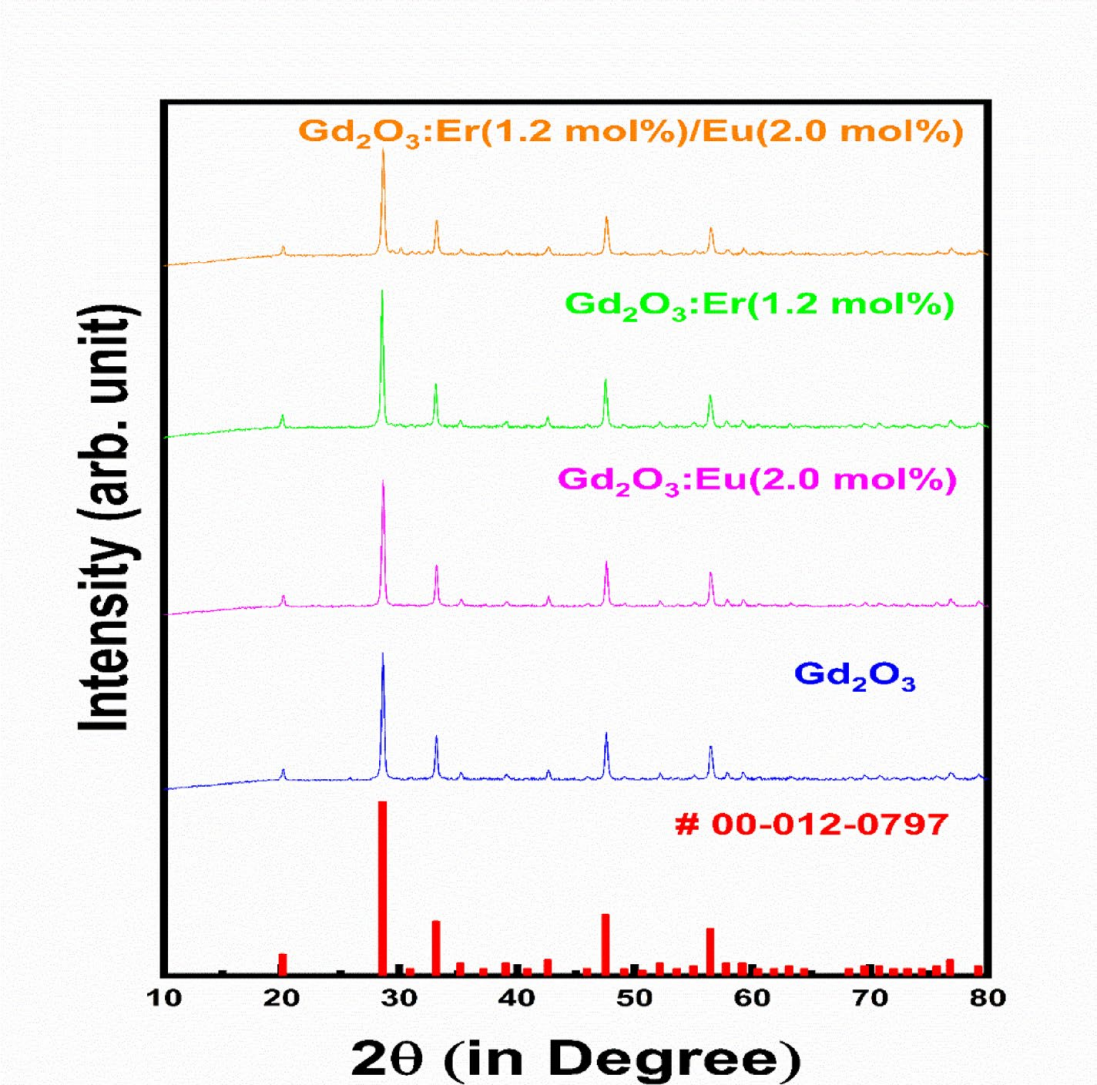
XRD Pattern of Gd_2_O_3_, Gd_2_O_3_:Eu, Gd_2_O_3_:Er, Gd_2_O_3_:Er/Eu with standard ICSD data.

XRD pattern of Gd_2_O_3_, Gd_2_O_3_:Eu (4.0 mol%), and Gd_2_O_3_:Er (1.2 mol %) is recorded because, in the PL study, they show maximum emission intensity. Regarding co-doped phosphor, Gd_2_O_3_:Er(1.2 mol %)/Eu(2.0 mol %) has maximum energy transfer efficiency. Above XRD pattern all peak of all prepared phosphor is matched with standard XRD data ICSD no: 00-12-0797 (Fig. 2). Adding impurities in host lattices such as Er and Eu does not contribute to the XRD pattern in the form of any extra peak. The absence of an impurity peak concludes that all impurities are incorporated into the host lattice without changing or adding any other phase in the prepared phosphor. Sharp peaks indicate the high crystallinity synthesized phosphor.

**Fig. 3:**
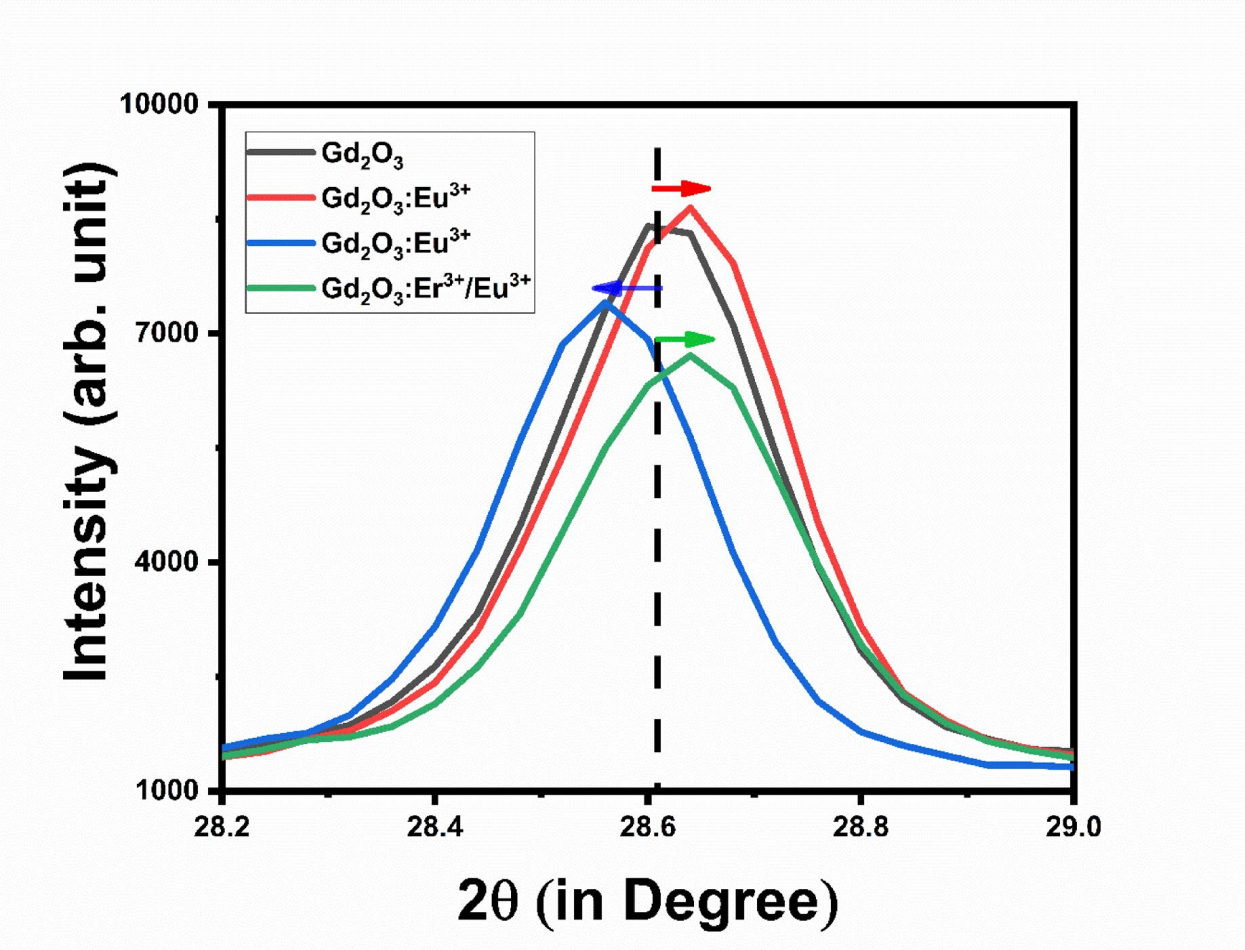
The XRD shift in of Gd_2_O_3_, Gd_2_O_3_:Eu, Gd_2_O_3_:Er, Gd_2_O_3_:Er/Eu.

Figure 3 shows the shift and change in intensity of host Gd_2_O_3_ on adding impurity. Adding a dopant like Eu (0.947 Å), which has a larger radius than host Gd (0.938 Å), creates lattice expansion in the host lattice expansion in host lattice that shift the XRD pattern toward small angle. When we add Er (0.890 Å) as dopant which has less radii leads to lattice contraction in host lattice that result shift in XRD pattern toward higher angle. Dopant Eu create the internal compressive strain, while internal tensile strain. Adding Er as dopant increases the crystallinity of prepared phosphor while adding Eu decreases the crystallinity. When both impurities are added to the host lattice, the difference in radii between Er and Gd is greater than that between Eu and Gd. So, shift in XRD pattern is primarily due the dopant Er so XRD pattern is shift toward higher angle. This XRD pattern has minimum intensity among all that indicate the decreasing crystallinity due lattice strain, dopant mismatch and defect formation. Using the Debye–Scherrer method, Table no. 1 shows the crystalline sizes of Gd_2_O_3_, Gd_2_O_3_:Eu^3+^, Gd_2_O_3_:Er^3+^, and Gd_2_O_3_:Er^3+/^ Eu^3+^. As impurities are added, the crystalline size of Gd_2_O_3_ decreases. Due to a considerable amount of strain generated in the lattice, the radius of the Eu ion is somewhat mismatched with that of the Gd, which is reflected in the crystalline size of the crystal. Because of lattice contraction, which results in high strain in the lattice, Er-doped Gd_2_O_3_ phosphor exhibits notable reductions in crystalline size. Gd_2_O_3_:Er^3+^/Eu^3+^ phosphor have coupled strain and increased defect density due to impurity, the crystalline size of Gd_2_O_3_:Er^3+^/Eu^3+^ reduces further.

**Table 1:** Crystalline size of Gd_2_O_3_, Gd_2_O_3_:Eu^3+^, Gd_2_O_3_:Er^3+^, and Gd_2_O_3_:Er^3+/^ Eu^3+^

Crystalline size using Debye–Scherrer method			
Gd_2_O_3_	Gd_2_O_3_:Eu^3+^	Gd_2_O_3_:Er^3+^	Gd_2_O_3_:Er^3+/^ Eu^3+^
83.92944 nm	79.94508 nm	73.19694 nm	68.48865 nm

**Fig. 4:**
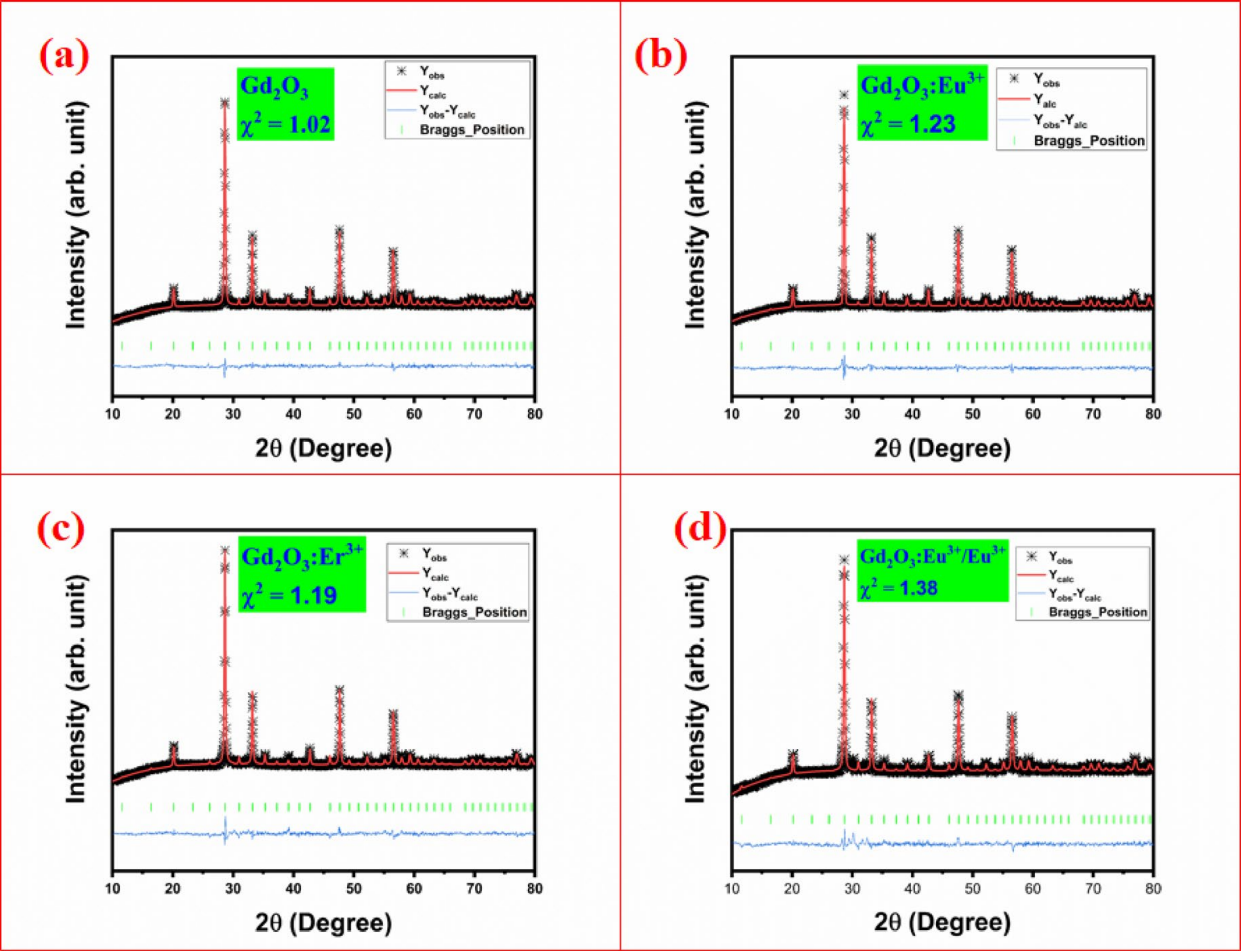
Rietveld refinement of Gd_2_O_3_ (**a**), Gd_2_O_3_:Eu^3+^ (**b**), Gd_2_O_3_:Er^3+^ (**c**), and Gd_2_O_3_:Eu^3+^/Er^3+^ (**d**).

To find the change in crystal structure and parameter of Gd_2_O_3_, Gd_2_O_3_:Eu, Gd_2_O_3_:Er, Gd_2_O_3_:Er/Eu we carried out the Rietveld refinement. Rietveld refinement is done with the help of full proof software by assuming the peak shape Pseudo-Voigt asprofile function with linear background. Parameters of Rietveld refinement is shown in Table 2. Lattice parameter after refinement is shown in Table 3 comparing with previous study with obtained data. Rietveld refinement of Gd_2_O_3_, Gd_2_O_3_:Eu, Gd_2_O_3_:Er, Gd_2_O_3_:Er/Eu shown in Fig. 4a–d, respectively. All prepared phosphor has cubic crystal structure with I 21 3 space group. Impurity does not change the crystal structure, it only affects the lattice parameter as shown in Table 3. In cubic structure a = b = c and α = β = γ so we only mention the a and α in table. Lattice parameter of Gd_2_O_3_ has nearly same as compare to previous study. When we add the impurity Eu it increases the lattice parameter a, and α is same. Incorporation of Er slightly reduce the lattice parameter a, and α is same. When we co-doped Er and Eu in lattice slightly reduction in size but less than Gd_2_O_3_:Er with α is same.

**Table 2:** Rietveld refinement parameter Gd_2_O_3_, Gd_2_O_3_:Eu^3+^, Gd_2_O_3_:Er^3+^, and Gd_2_O_3_:Er^3+/^ Eu^3+^

Sr no	Name	R_p_	R_wp_	R_exp_	*χ*^*2*^
1	Gd_2_O_3_	26.5	15.2	15.07	1.02
2	Gd_2_O_3_:Eu(4.0 mol%)	32	16.8	15.17	1.23
3	Gd_2_O_3_:Er (1.2 mol %)	34.3	18.5	16.92	1.19
4	Gd_2_O_3_:Er(1.2 mol %)/Eu(2.0 mol %)	34.8	20.2	17.2	1.38

**Table 3:** Lattice parameter of Rietveld refinement parameters Gd_2_O_3_, Gd_2_O_3_:Eu^3+^, Gd_2_O_3_:Er^3+^, and Gd_2_O_3_:Er^3+/^ Eu^3+^

Sr no	Name	Gd_2_O_3_	Gd_2_O_3_:Eu(4.0 mol%)	Gd_2_O_3_:Er (1.2 mol %)	Gd_2_O_3_:Er(1.2 mol %)/Eu(2.0 mol %)	Standard^21^
1	a	10.799713	10.806700	10.793955	10.796558	10.722
2	α	90^°^	90^°^	90^°^	90^°^	90^°^
3	Volume	1259.612	1262.058	1257.598	1258.508	1232.7

**Fig. 5:**
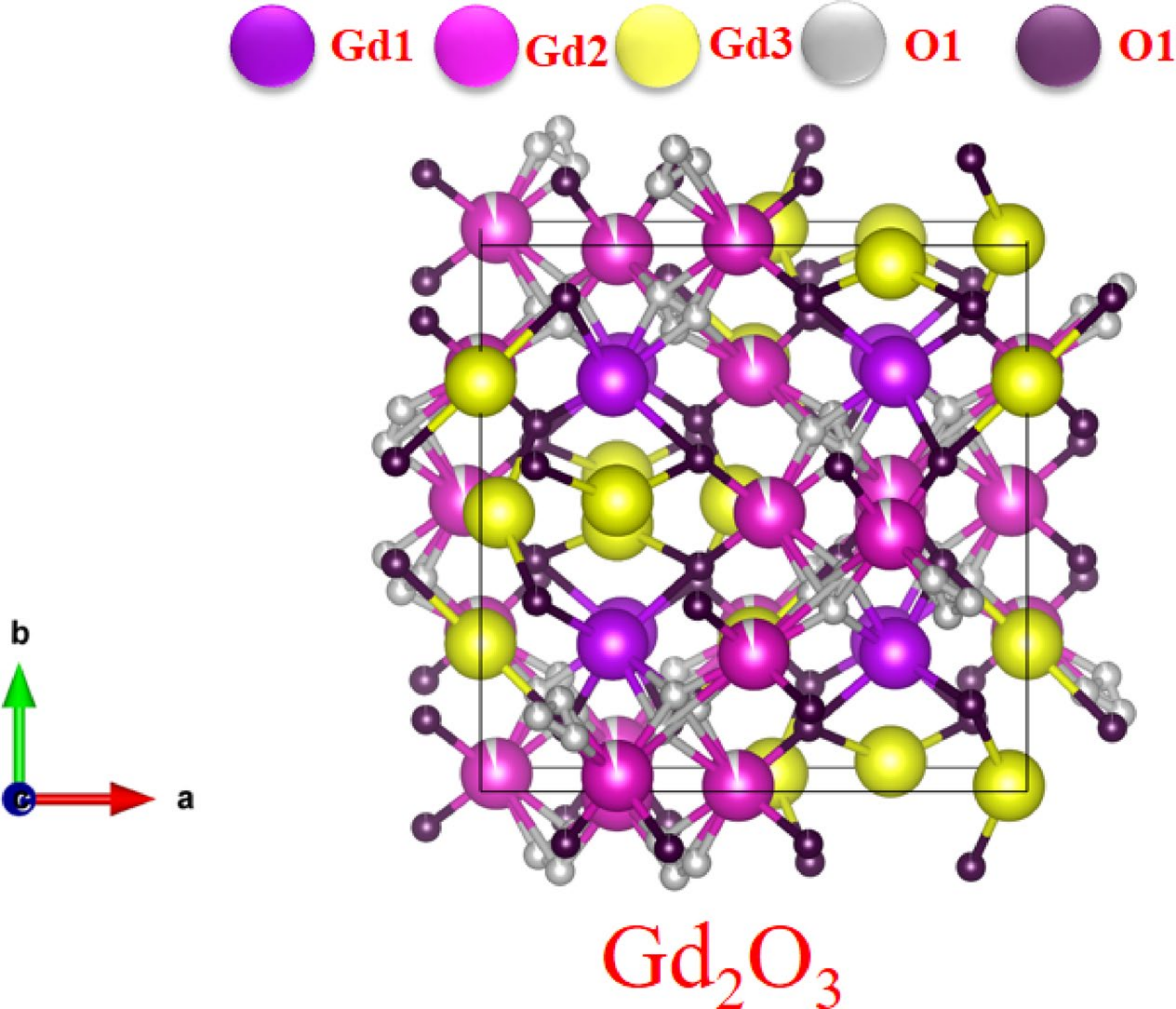
Crystal structure of Gd_2_O_3_.

The crystal structure of Gd₂O₃ is cubic and belongs to space group I 21 3 as shown in Fig. 5. This crystal structure contains two unique O^2-^ sites and three cryptographically distinct sites of Gd^3+^. Three distinct Wyckoff positions are occupied by each Gd^3+^ ion. Every Gd^3+^ ion forms a connection with six oxygen atoms to create GdO₆ octahedra, which share their corners and edges with nearby polyhedral. These create a moderately distorted 3D framework. The length of the bond varies from 1.96507 Å to.

2.9312 Å. The OGd_4_ tetrahedron is created when one O^2−^ ion coordinates with four Gd^3+^ ions. OGd_6_ octahedra are formed when the second O^2−^ ion coordinates with six Gd^3+^ ions. The octahydra’s till angle between them is ranges from 15 to 45°, and OGd4 and OGd_6_ share corners and edges. Each unit cell of Gd_2_O_2_ contains a total of 40 atoms, comprising 16 Gd^3+^ ions occupying three distinct crystallographic sites, and 24 O^2−^ ions distributed over two inequivalent oxygen positions.

**Fig. 6:**
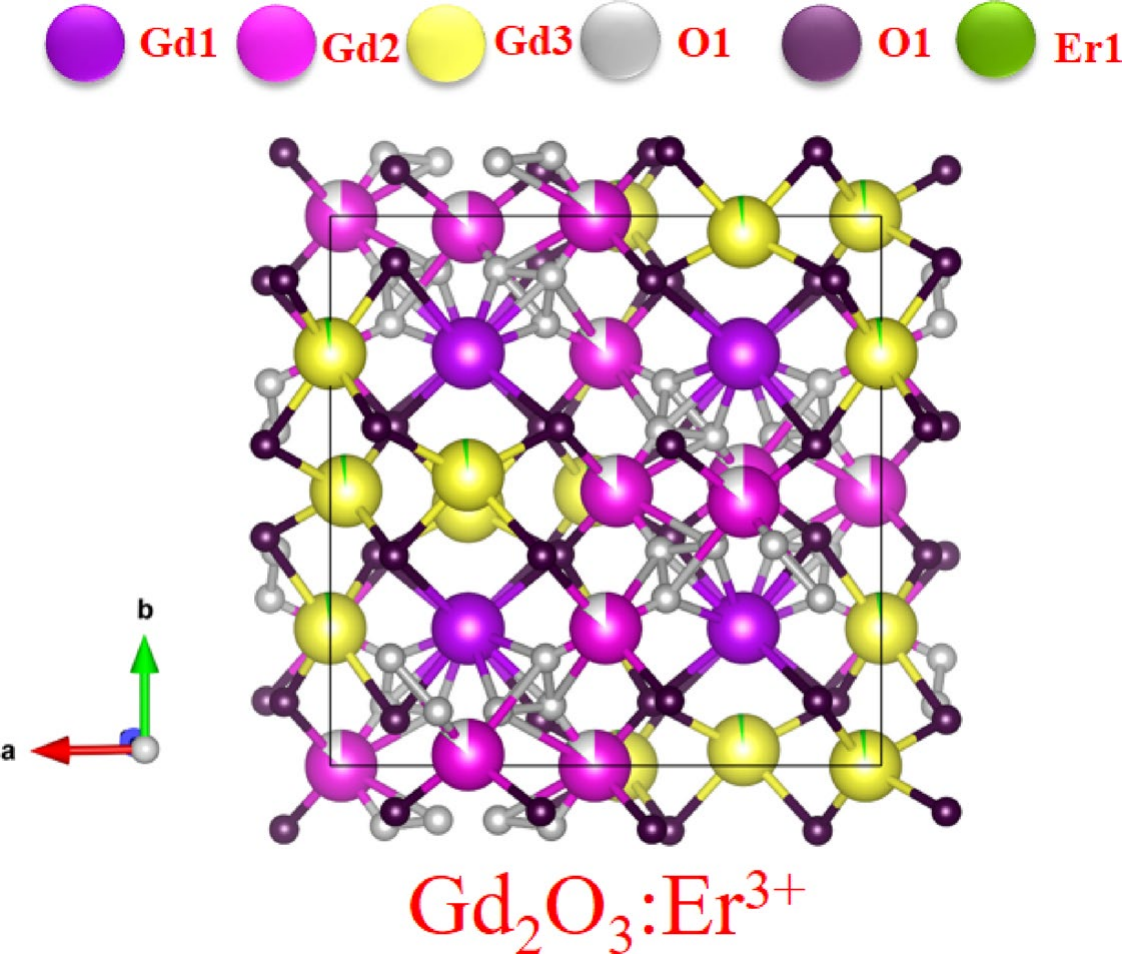
Crystal structure of Gd_2_O_3_:Er^3+^

Figure 6 shows the crystal structure of Gd_2_O_3_:Eu^3+^. Addition of Er does not affect the crystal structure significantly. Er doped Gd_2_O_3_ have also same cubic structure and space symmetry as Gd_2_O_3._ Er ion can replace the Gd ion in structure due nearly same radius and same + 3 oxygen state. Due to charge balance it allow the doped structure with same framework with some distortion. On Rietveld refinement analysis we can conclude that the Er ion is mostly substitute at place of Gd3 ion in crystal structure as in Fig. 6 with green color. The length of the bond varies from 1.96507 to 2.9312 Å.

**Fig. 7:**
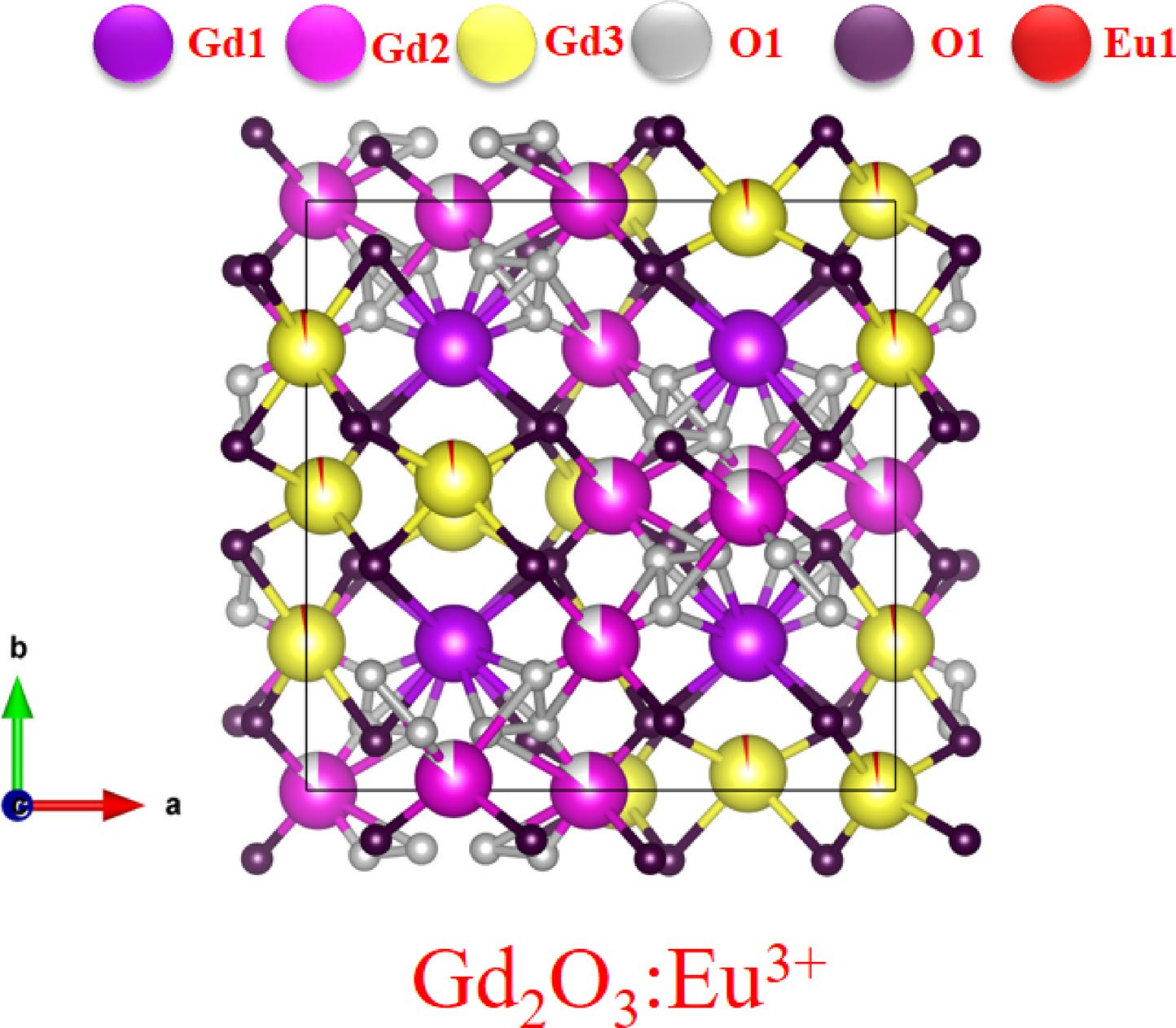
Crystal structure of Gd_2_O_3_:Eu^3+^

Figure 7 shows crystal structure of Gd_2_O_3_:Eu^3+^. When Eu is dopant in Gd_2_O_3_ due to charge neutrality and comparable radii framework remain same. There have some change in bond length varies from to 1.9 to 2.54 Å. On comparing the position of ion after Rietveld refinement we can conclude that Eu ion is replaced in the place of Gd^3+^ ion. Shown in Fig. 7 in red color.

**Fig. 8:**
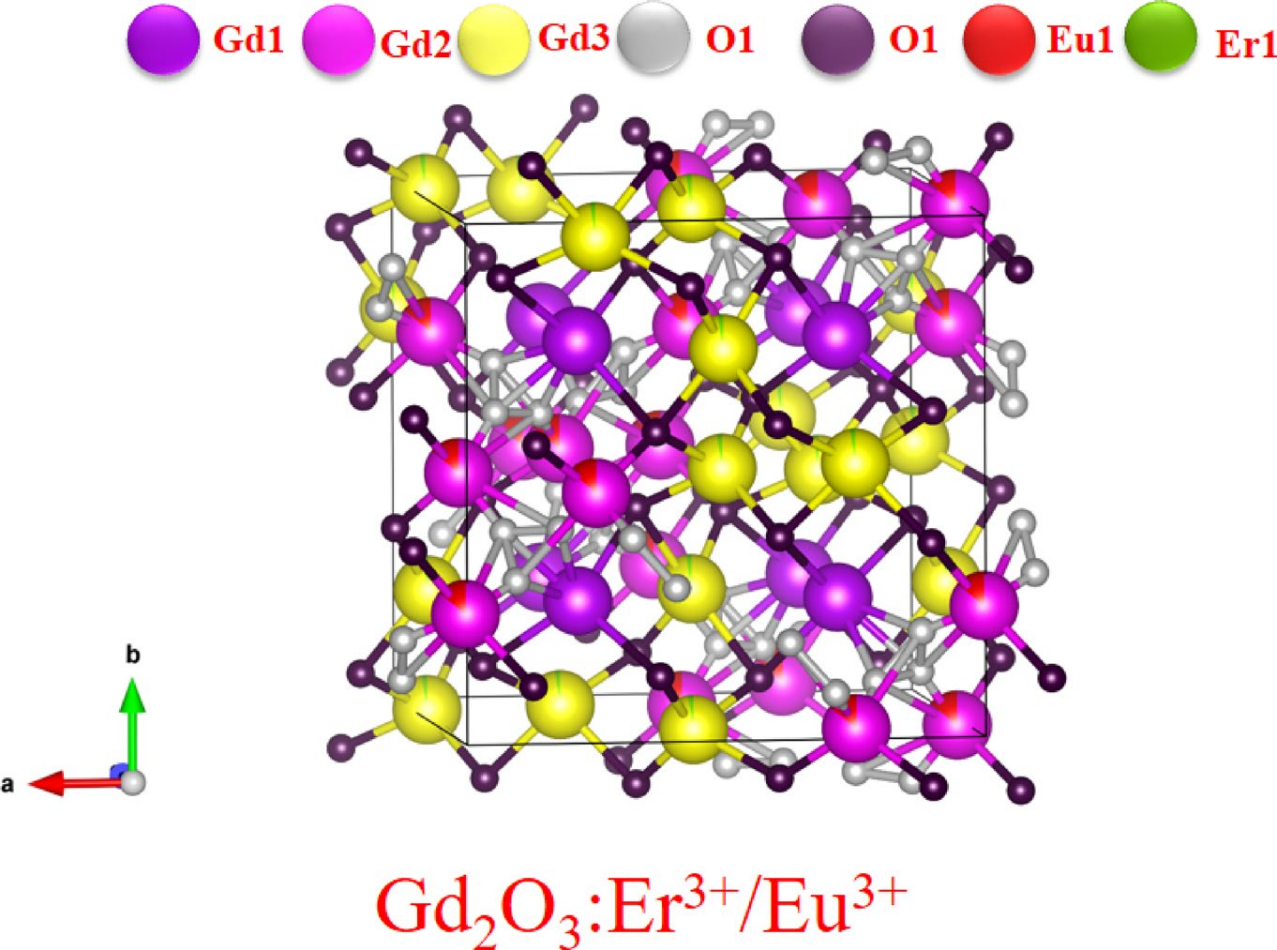
Crystal structure of Gd_2_O_3_:Eu^3+^/Er^3+^.

Figure 8 shows the crystal structure of Gd_2_O_3_:Eu^3+^/Er^3+^ Adding both Er and Eu ion does not change the cubic structure of crystal due to same + 3 oxidation state and comparable atomic radius. Er and Eu have 3 different site replace and 2 different O^2−^ site to make bond. Bond length varies from 2.2 to 2.76 Å. Which ion is substitute and in which place it depends on the local coordination geometry and synthesis conditions. Substitution causes the distortion in crystal structure in lattice. Different radii cause different bond length and co-ordinate angle with in GdO_6_ octahedral. The position of ion in lattice is shown in Table 4.

**Table 4:** Position of atom in Gd_2_O_3_, Gd_2_O_3_:Eu^3+^, Gd_2_O_3_:Er^3+^, and Gd_2_O_3_:Er^3+/^ Eu^3+^.

Sr no	Name	Element	X	Y	Z
1	Gd_2_O_3_	Gd1	0.25815	0.25815	0.25815
		Gd2	0.02886	0.00000	0.25000
		Gd3	0.53332	0.00000	0.25000
		O1	0.17170	− 0.10548	0.25815
		O2	0.09962	0.39717	0.39717
2	Gd_2_O_3_:Eu^3+^	Gd1	0.25814	0.25814	0.25814
		Gd2	0.02656	0.00000	0.25000
		Gd3	0.53348	0.00000	0.25000
		O1	1.10640	1.13268	1.16555
		O2	0.09184	0.39296	0.34181
		Eu1	0.53348	0.00000	0.25000
3	Gd_2_O_3_:Er^3+^	Gd1	0.25276	0.25276	0.25276
		Gd2	0.02917	0.00000	0.25000
		Gd3	0.51575	0.00000	0.25000
		O1	0.21024	0.15593	0.43926
		O2	0.08416	0.38257	0.34608
		Er1	0.51575	0.00000	0.25000
4	Gd_2_O_3_:Er^3+^/Eu^3+^	Gd1	0.25108	0.25108	0.25108
		Gd2	0.01685	0.00000	0.25000
		Gd3	0.52838	0.00000	0.25000
		O1	21.22171	14.93350	14.37767
		O2	0.08601	0.38535	0.38099
		Er1	0.01685	0.00000	0.25000
		Eu1	0.52838	0.00000	0.25000

### Scanning electron microscope (SEM)

**Fig. 9:**
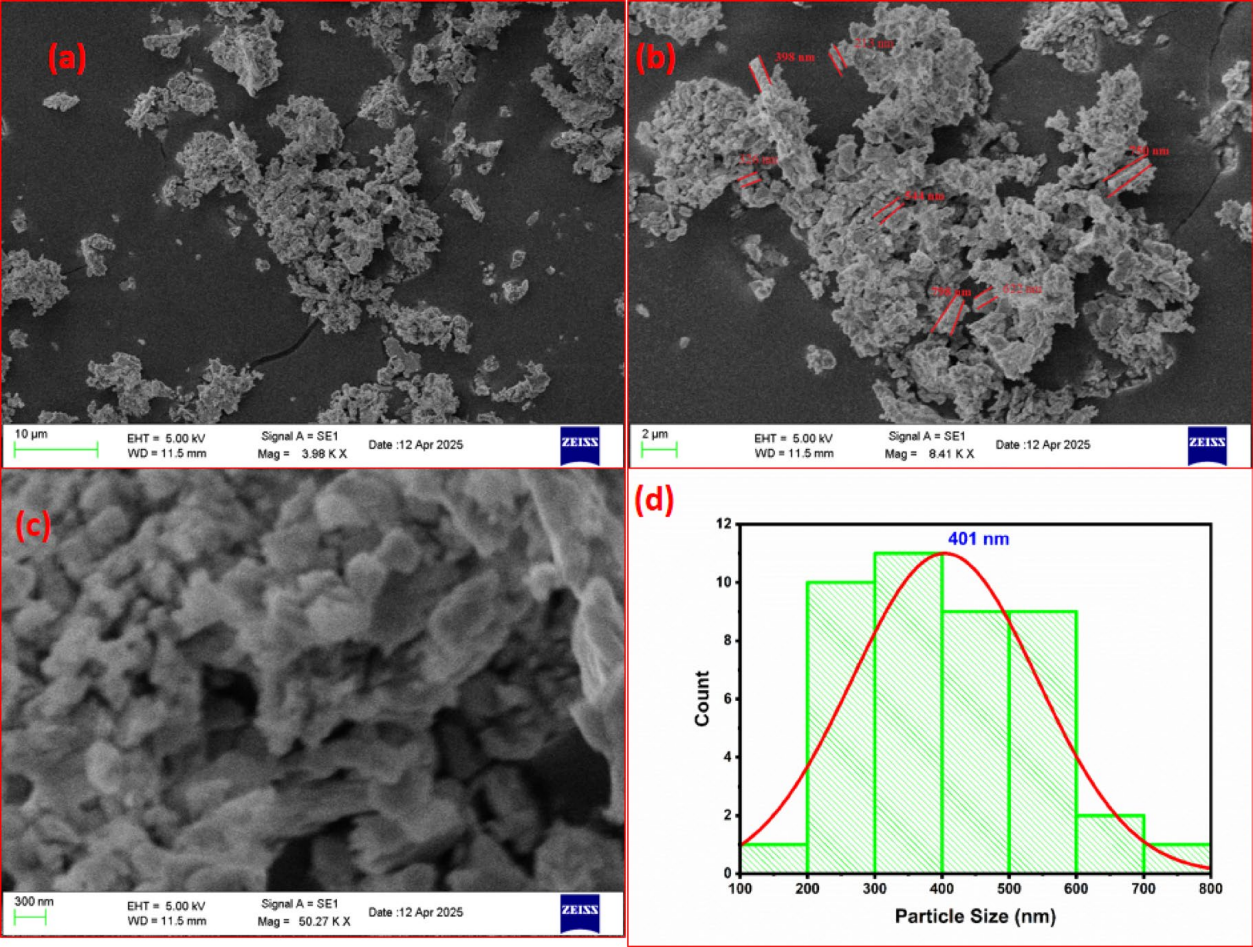
SEM image of Gd_2_O_3_ morphology and particle size distribution.

The morphology study of the prepared sample is carried out using SEM analysis. Figure 9a–c shows SEM images of Gd_2_O_3_ at 10 µm, 2 µm, and 300 nm respectively. The prepared phosphor is sintered at 800 °C therefore particle seem to agglomerate. Particles are in the micrometer range. During the process of sintering gases are released from the prepared sample, which makes it more porous in nature. Particles had irregular sizes due to uncontrolled heating during the sintering process. Particles have rod-like structures, and the width of some particles are marked in Fig. 9b. Porous nature of prepared phosphor is very useful in solid-state lighting applications. Figure 9d shows a particle size distribution curve ranging from 100 to 800 nm with an average particle size of 401 nm. The porous and irregular morphology of rare-earth-doped particles enhances photon absorption due to increased surface area, improving PL emission. However, excessive agglomeration introduces surface defects and scattering losses, which can quench luminescence intensity^22^.

### Transmission electron microscopy (TEM)

**Fig. 10:**
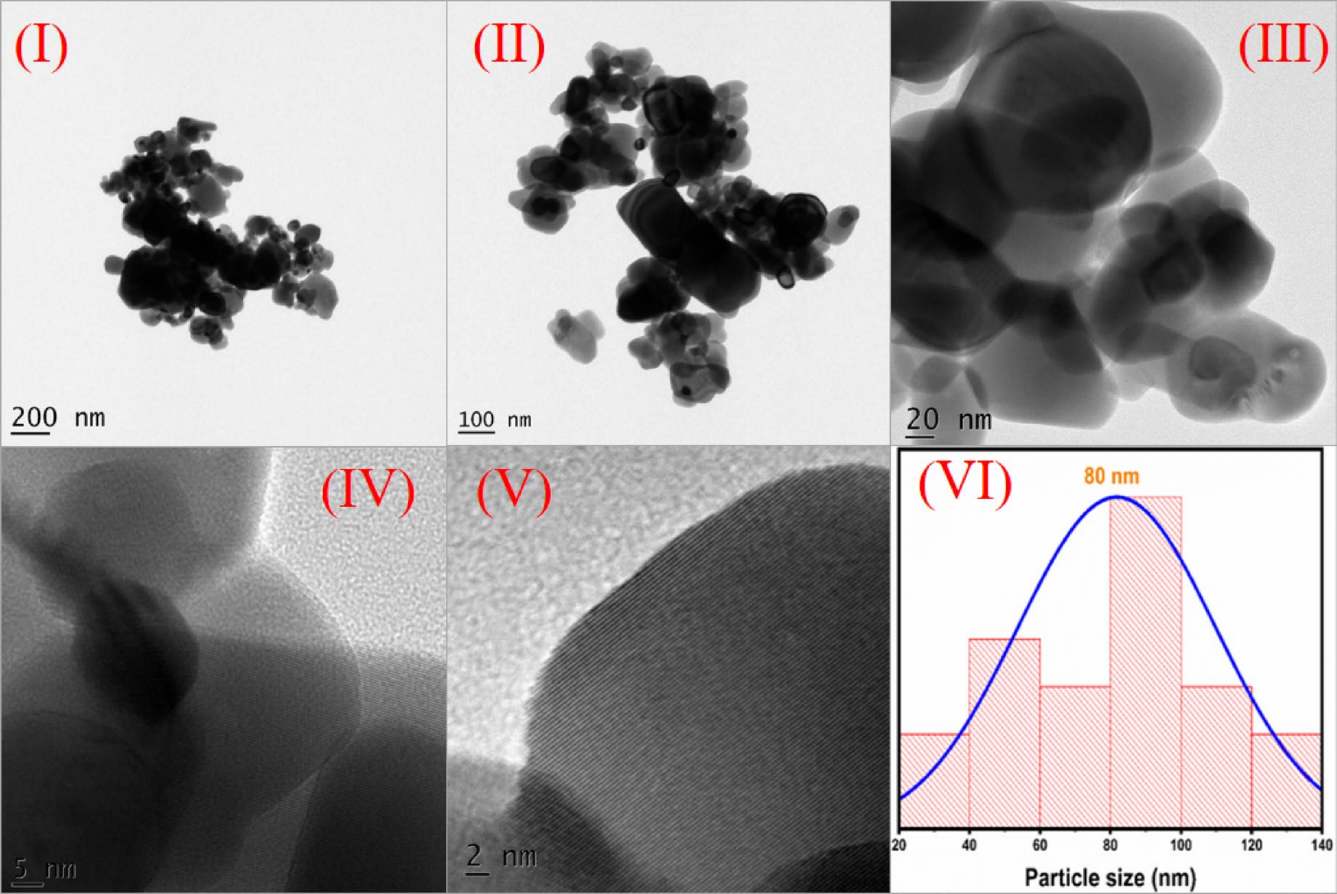
TEM image (I–V) and particle size distribution of Gd_2_O_3_.

Figure 10I, II, III, IV, and V shows TEM image of Gd_2_O_3_ at 200 nm, 100 nm, 20 nm, 5 nm, and 2 nm respectively. TEM images shows irregular shapes. Some particles have spherical shape. The TEM images (IV, V) reveal well-defined lattice fringes, confirming crystalline behavior of the synthesized nanoparticles confirmed by XRD study. Figure 10VI shows particle size distribution curve of TEM. Particle size of prepared sample is ranges from 20 to 140 nm with average particle size of 80 nm. The particle size distribution exhibits a Gaussian profile, indicating a predominantly uniform particle size with minor variations.

### Fourier transform infrared spectroscopy (FT-IR)

**Fig. 11:**
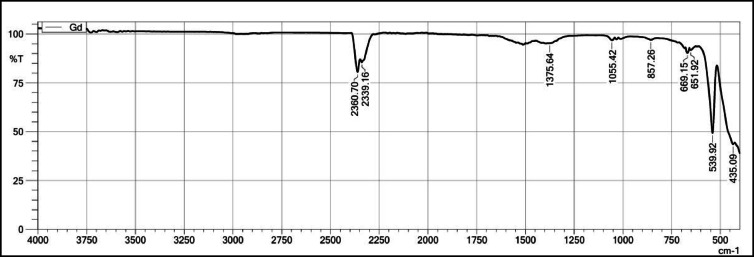
FT-IR Spectrum of Gd_2_O_3_.

The FT-IR spectra of Gd_2_O_3_, Gd_2_O_3_:Eu^3+^(2.0 mol%), Gd_2_O_3_:Er^3+^ (1.2 mol%), and Gd_2_O_3_:Eu^3+^ (2.0 mol%)/ Er^3+^(1.2 mol%) were studied. These concentrations of Eu and Er were selected because they have maximum intensity at that concentration while in context with Gd_2_O_3_:Eu^3+^ (2.0 mol%)/ Er^3+^(1.2 mol%) it has maximum energy transfer happening. FT-IR spectra are recorded from 400 to 4000 cm^−1^. Figure 11 shows the FT-IR spectra of Gd_2_O_2_. Spectra contain 2 major peaks at 435.09 and 539.92 cm^−1^ and minor peaks at 857.26 cm^−1^, 1055.42 cm^−1^, 1375.64 cm^−1^, 2339.16 cm^−1^, and 2360.70 cm^−1^. Peaks at 435.09, 857.26 cm^−1^, and 539.92 cm^−1^ are due to vibration between the metal and oxygen. This spectrum is due to the Gd-O vibration. These strong peaks indicate the formation of Gd₂O₃. A peak at 1375.64 cm^−1^ occurs due to C-H bending or NO_2−_ vibration that indicates the presence of the NO_3_^−^ group. On comparing the intensity of these peaks with other peaks, it indicates the presence of this group is minimal and the presence of a precipitating agent. The peak at 2339.16 cm^−1^ and 2360.70 cm^−1^ is due to the C-O vibration. These peaks indicate the host has a tendency to absorb the CO₂ from the environment because in the precursor there is an absence of compounds that contribute to C-O compounds. In these spectra, there is an absence of a peak near 3400 cm^−1^ that indicates the compound does not absorb the moisture from the surrounding^23–27^.

**Fig. 12:**
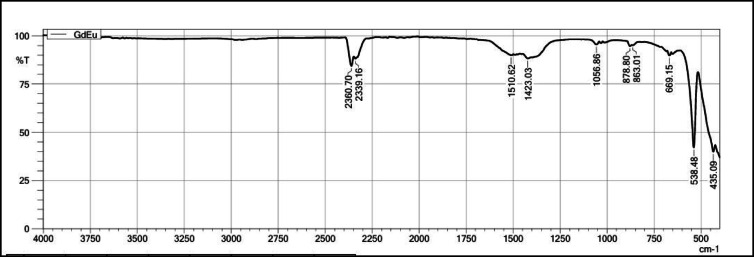
The FT-IR Spectrum of Gd_2_O_3_:Eu^3+^.

Figure 12 shows FT-IR spectra Gd_2_O_3_:Eu^3+^ recorded from 4000 to 400 cm^−1^. These spectra also contain peaks at 435.09 cm^−1^, 538.48 cm^−1^, 699.15 cm^−1^, and 878.80 cm^−1^ due to Gd-O and Eu–O bond present in the host. The peaks at 1423.03 cm^−1^ and 1510.62 cm^−1^ are due presence of C–H bending or NO_3_^−^ vibration. The peaks at 2360.70 cm^−1^ and 2339.16 cm^−1^ are due to the presence of C–O vibration in the host lattice. Vibration due –OH is absent which indicates no such moisture is absorbed surroundings after the dopant is introduce in lattice.

**Fig. 13:**
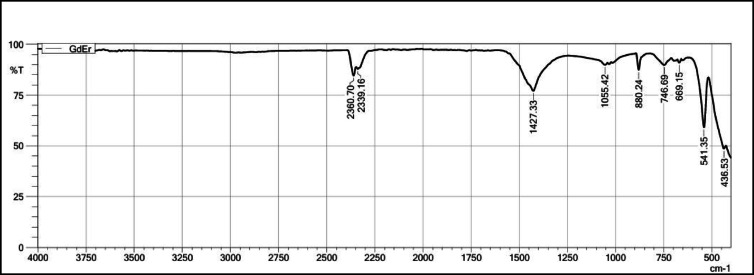
The FT-IR Spectrum of Gd_2_O_3_:Er^3+^

Figure 13 shows FT-IR spectra of Gd_2_O_3_:Er^3+^. Spectrum contains peaks at 436.53 cm^−1^, 541.35 cm^−1^, 699.15 cm^−1^, 746.69 cm^−1^, and 880.24 cm^−1^ due to Gd-O and Er-O vibration in the lattice. The peak at 1427.33 cm^−1^ is due to the presence of C–H bending or NO−⁻ vibration. The peak at 2360.70 cm^−1^ and 2399.16 cm^−1^ is due to C–O vibration. Like above this lattice does not have vibration due to –OH.

**Fig. 14:**
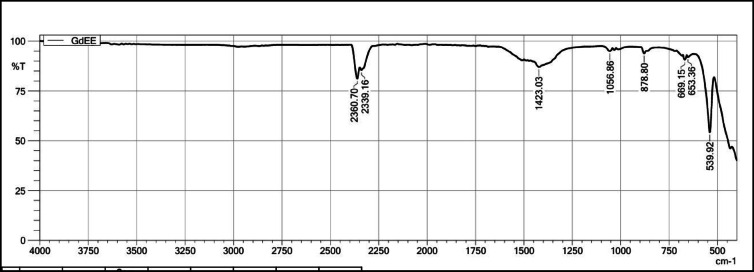
The FT-IR Spectrum of Gd_2_O_3_: Er^3+^/Eu^3+^.

Figure 14 shows the FT-IR spectra of Gd_2_O_3_:Er^3+^/Eu^3+^. Spectra contains peaks at 436.53 cm^−1^, 539.92 cm^−1^, 653.36 cm^−1^, 669.15 cm^−1^, and 878.80 cm^−1^ due to vibration of Gd-O, Eu–O, and Er-O bonds present in the host lattice. The peak at 1423.03 cm^−1^ is due to the NO^3−^ group present in the lattice. Peak on 2360.70 cm^−1^ and 2339.16 cm^−1^ is due to absorption of CO_2_ from surrounding. The absence of a peak near 3400 cm^−1^ indicates the absence of an –OH group in the lattice.

On comparing all four FT-IR spectra, all spectra have major peaks due to Gd-O vibration that confirm the formation of Gd_2_O_3_. The intensity of peaks around 653 cm^−1^, 669 cm^−1^, and 878 cm^−1^ is more as compared to pure Gd_2_O_3_­that might indicate the vibration between Eu–O and Er–O. The Gd_2_O_3_:Er^3+^ has a maximum peak intensity of around 1422 cm^−1^. All FT-IR spectra contain peak around 2360 cm^−1^ due to vibration of C-O band. All FT-IR spectra do not have any peak near 3400 cm^−1^ due to the absorption of moisture from the surroundings. That indicates that adding a dopant does not affect the chemical nature of the host lattice. Absorbing the moisture leads to oxides into hydroxide that affect the chemical and physical properties of the host when we compare to La_2_O_3._ When La_2_O_3_ encounters moisture, the La_2_O_3_ becomes La(OH)_3_ and due to this PL property decreases significantly, and red-emitting phosphor becomes white emitting phosphor. This property of this host can be very useful in barcode and fingerprint application where the phosphor comes with direct contact of moisture.

### Photoluminescence (PL)

**Fig. 15:**
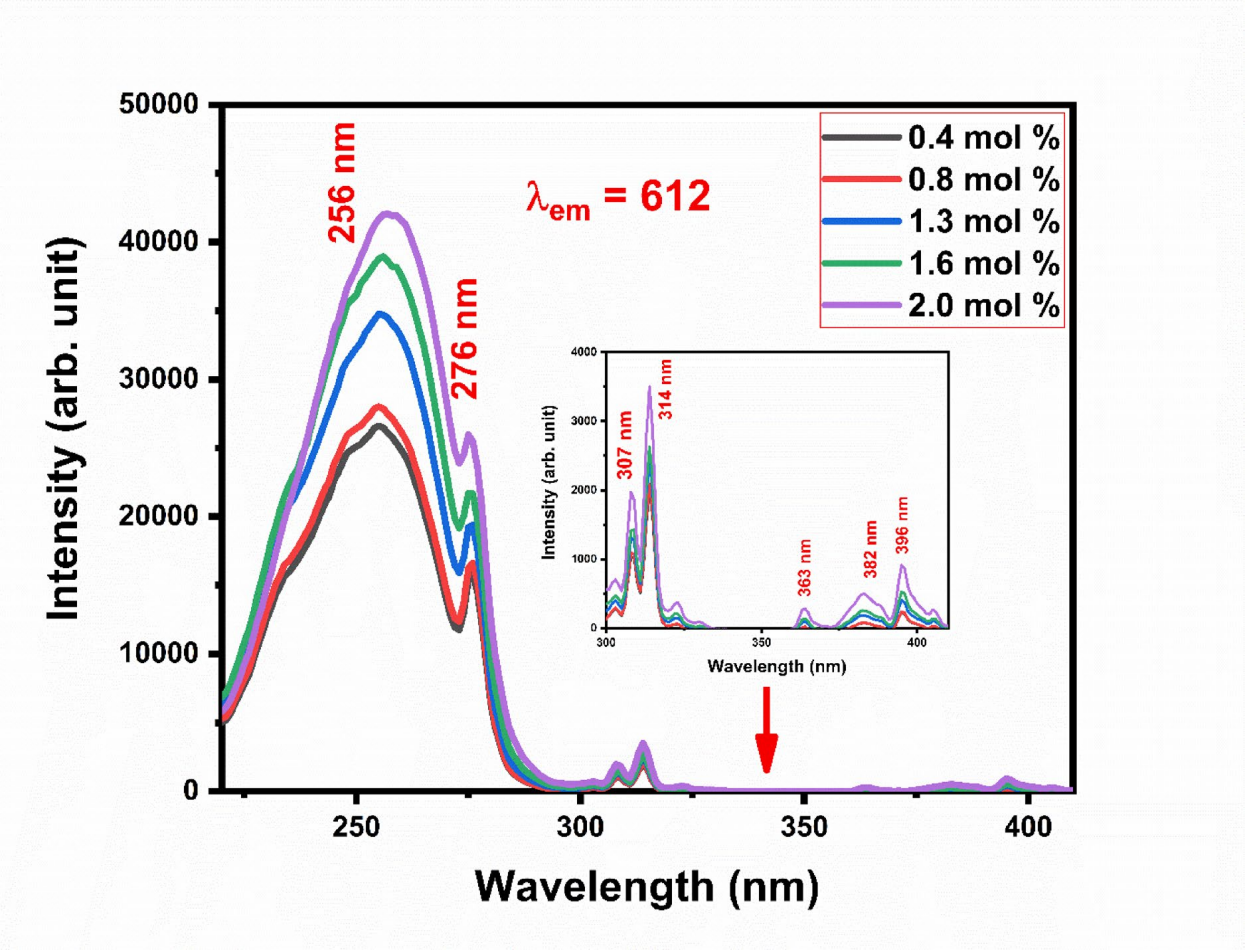
The excitation spectra of Gd_2_O_3_:Eu^3+^ at emission wavelength 612 nm.

Figure 15 shows the excitation spectra Gd_2_O_3_:Eu^3+^ at emission wavelength 612 nm at different concentrations of Eu ion such as 0.4–2.0 mol%. The excitation spectra have one broad and intense peak centered at 256 nm. This peak is due to charge transfer between Eu^3+^ and O^2−^. Broad nature of the peak is due to the wide range of O^2−^ sites present in the host lattice with Eu^3+^ having an inhomogeneous host lattice present in the lattice. Gd_2_O_3_ has a cubic crystal structure with 3 different cation sites. Gd ions have significant absorption in the range of 270–280 nm (near UV region) and formed peak at 276 nm. This absorption is due to the allowed interconfigurational transition in Gd^3+^ from the 4f.^7^ (^8^S_7/2_) (ground state) to 4f.^6^5d^1^ (excited state). This peak is weak as compared to the charge transfer band due to fewer sites contributing to this peak. This acts as a sensitizer for Eu^3+^ and transfers the energy to Eu to enhance the emission. Along with the above two peaks, excitation spectra contain peaks at 307 nm, 314 nm, 363 nm, 382 nm, and 396 nm. The peak at 307 nm is due to the transition^8^S_7/2_ → ^6^P_7/2_ of Gd. The sharp nature of the peak is due to the shielding of the 4f. electron configuration. The peak at 314 is due to ^8^S_7/2_ → ^6^I_7/2_ transition in Gd^3+^ ions. The intensity of the peak centered at 314 nm is more than the peak at 307 nm due to the ^8^S_7/2_ → ^6^P_7/2_ transition being more prominent with ^5^D₀ ground level of Eu^3+^^17,23–25^. Along with ^8^S_7/2_ → ^6^P_7/2_ transition of Gd^3+^ ion overlaps with transition ^5^D_o_ → ^5^D_3_ or higher transition of Eu^3+^. Gd_2_O_3_ has two district cation sites (C_2_ and S_6_ symmetries) in our case 314 nm is more prominent that indicate in host lattice Eu in one of the sites has more substitution that enhances the absorption in the host lattice. Strong sensitizer transition helps to increase the emission. The peak centered at 363 nm, 382 nm, and 396 nm is due to the^7^F_0_→ ^5^D_4_,^7^F_0_ → ^5^G_2_ and ^7^F_0_→ ^5^L_6_ transition of Eu, respectively^6,26–28^. In Gd_2_O_2_:Eu^3+^(2.0 mol%)phosphor, maximum absorption is observed as shown in Fig. 14. Since emission intensity is directly governed by the absorbed photons, this results in the strongest luminescence output^29^.

**Fig. 16:**
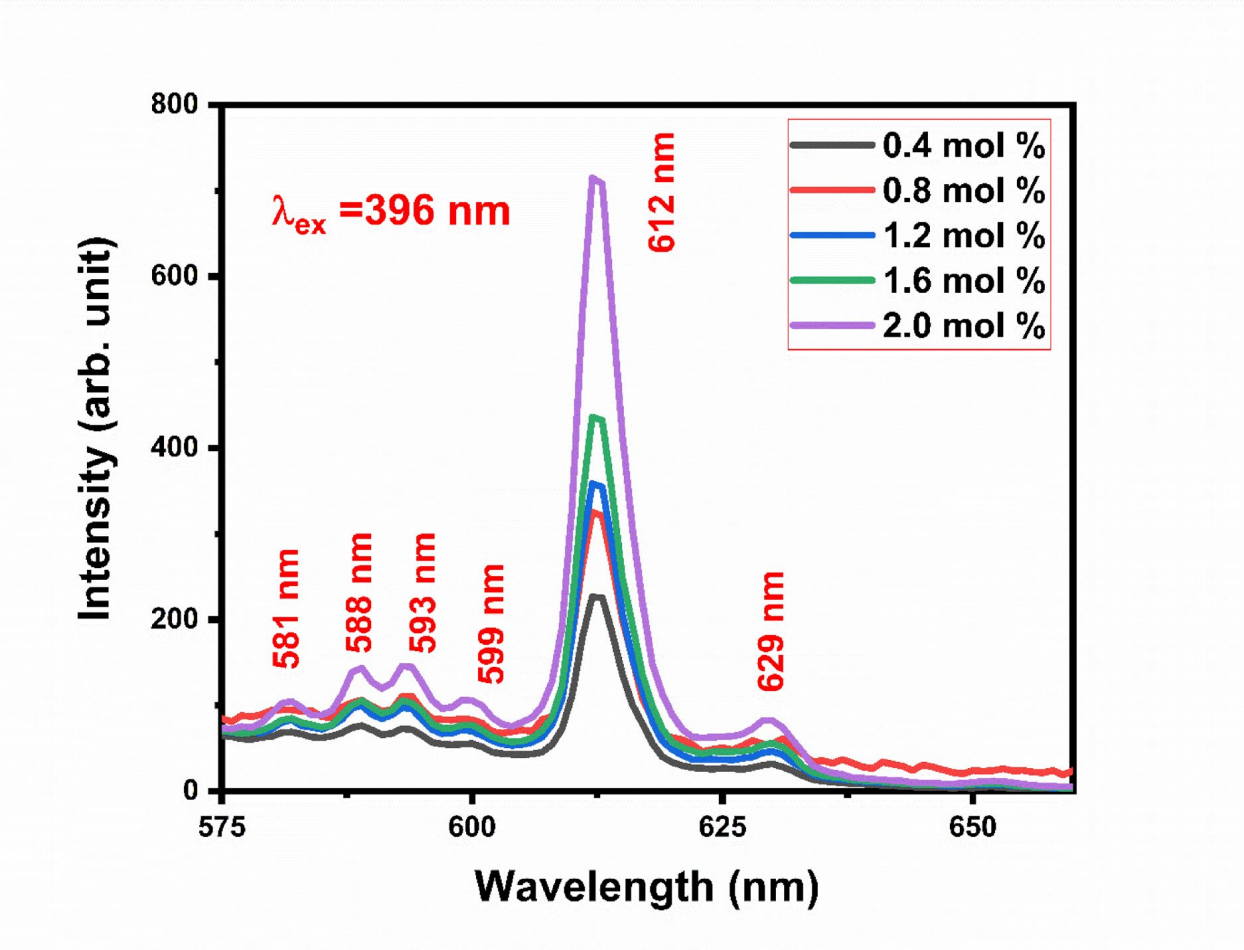
The emission spectra of Gd_2_O_3_:Eu at excitation wavelength 396 nm.

**Fig. 17:**
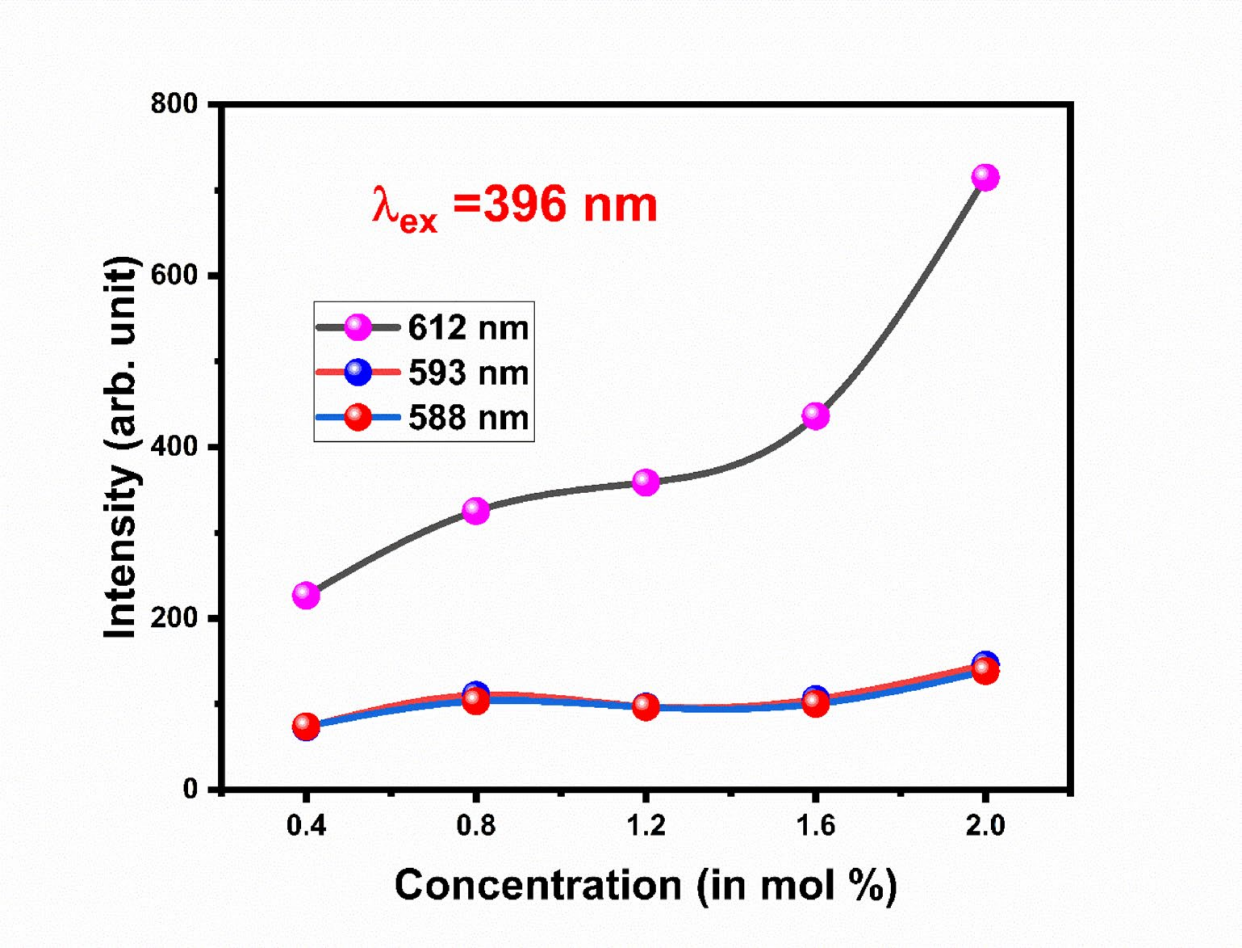
Concentration quenching of Gd_2_O_3_:Eu^3+^.

Gd_2_O_3_:Eu^3+^ phosphor is triggered by excitation wavelength at 396 nm shown in Fig. 16. Emission spectra are recorded from 575 to 675 nm. The emission spectra contain the one major peak centered at 612 nm due to ^5^D_0_ → ^7^F_2_ transition. Some minor peaks at 581 nm, and 629 nm are due to ^5^D_1_ → ^7^F_2_, and ^5^D_0_ → ^7^F_3_ transition respectively. The emission peaks at 588 nm and 593 nm correspond to the ^5^ D_0_ → ^7^F_1_ransition of Eu^3+^ ions^19^. The presence of two distinct peaks is attributed to the fact that Eu^3+^ ions can occupy different crystallographic sites in the Gd_2_O_2_ host lattice, leading to variations in the local environment and resulting in peak splitting. Emission spectra are recorded for different concentrations of Eu of 0.4 mol%, 0.8 mol%,1.2 mol%, 1.6 mol%, and 2.0 mol%. Figure 17 contains the emission intensity at 588 nm, 593 nm, and 612 nm with the varying concentration of dopant. Emission spectra of Gd_2_O_3_:Eu^3+^ at excitation wavelengths 314 nm and 256 nm are shown in Figs. S1 and S2 in supplementary data respectively^30–34^.

**Fig. 18:**
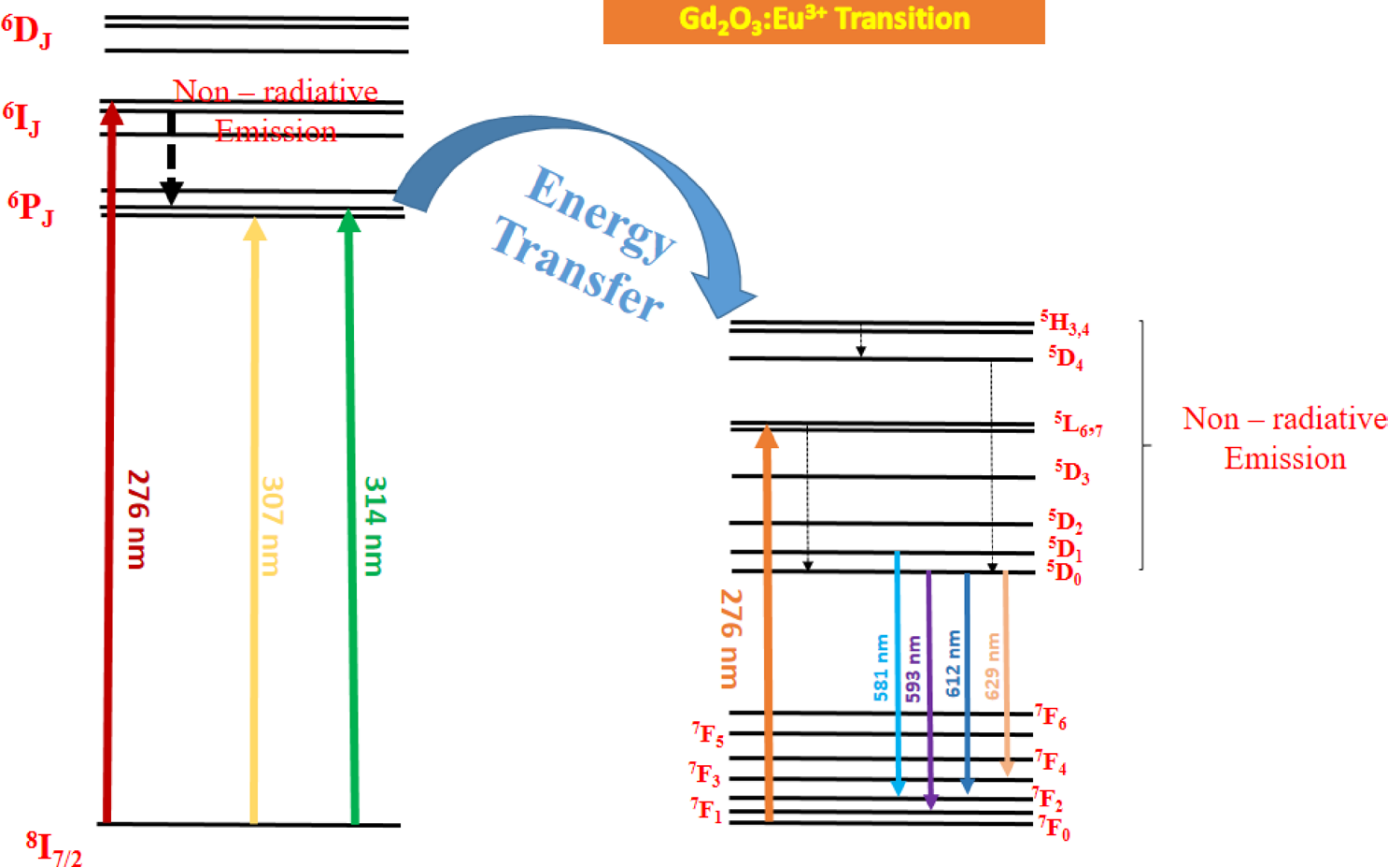
Energy level diagram of Gd_2_O_3_:Eu^3+^.

The detailed energy transitions of Gd_2_O_3_:Eu^3+^ shown in Fig. 18**. **Figure 18 contain the energy transfer mechanism in terms of energy level from Gd^3+^ ion to Eu^3+^ ion.

**Fig. 19:**
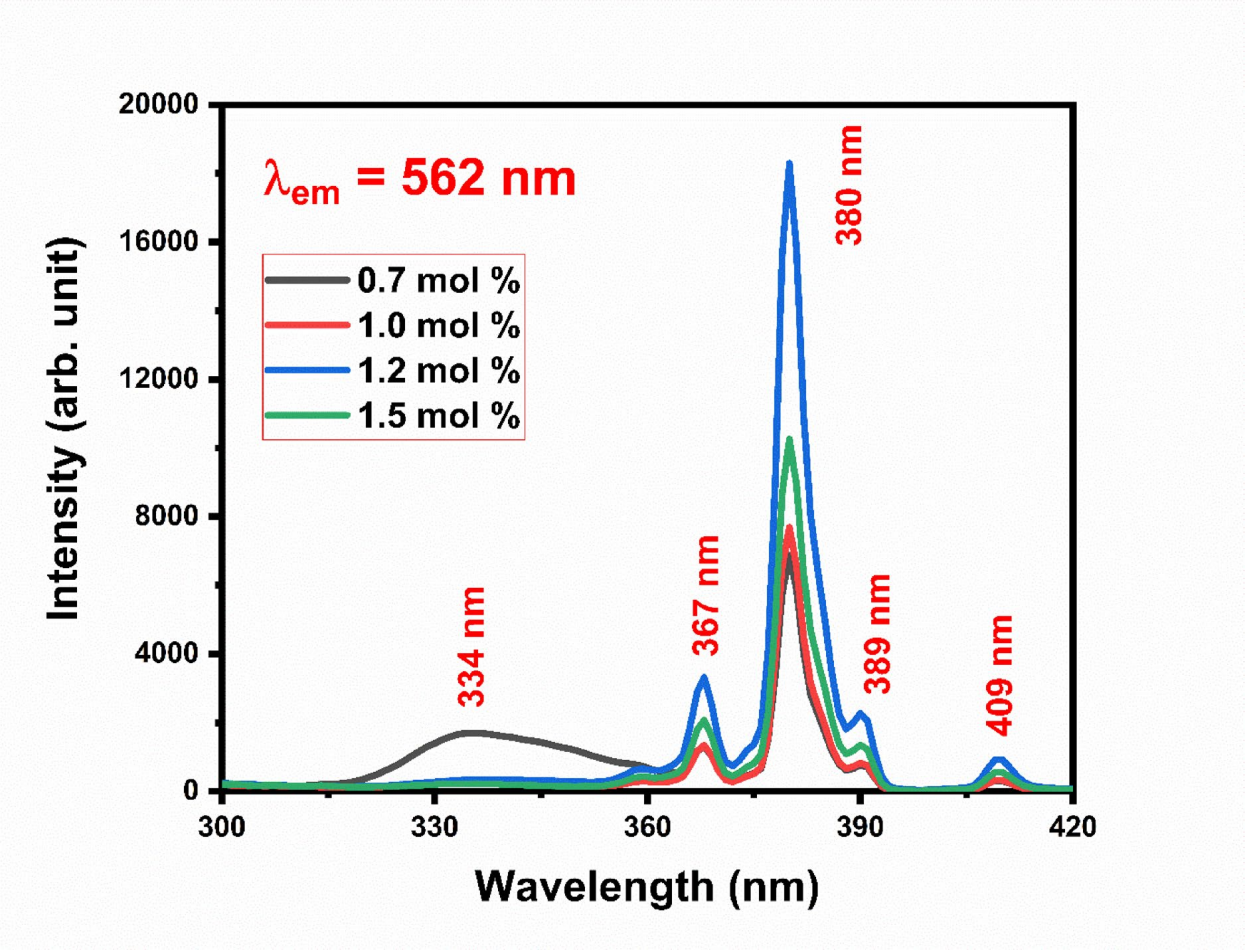
The excitation spectra of Gd_2_O_3_:Er^3+^ at emission wavelength 562 nm.

Figure 19 shows the excitation spectra of Gd_2_O_3_:Er^3+^ at concentrations of 0.7 mol%, 1.0 mol %, 1.2 mol%, and 1.5 mol%. The excitation spectra do not show a charge transfer band or peaks due to transitions in the host Gd lattice.These spectra have one major peak centered at 380 nm due to ^4^I_15/2_ → ^4^G_11/2_ transition with 3 minor peaks at 367 nm, 389 nm, and 409 nm due ^4^I_15/2_ → ^2^P_1/2_, ^4^I_15/2_→ ^2^H_9/2_, and ^4^I_15/2_→ ^4^F_7/2_. One board peak centered at 334 nm is observed only at Gd_2_O_3_:Er^3+^ (0.7 mol %)^35,36^. In Gd_2_O_2_:Er^3+^ phosphor, strong absorption is also observed in the excitation region. The absorbed photons enhance the characteristic green emission, confirming that higher absorption leads to more intense luminescence^29^.

**Fig. 20:**
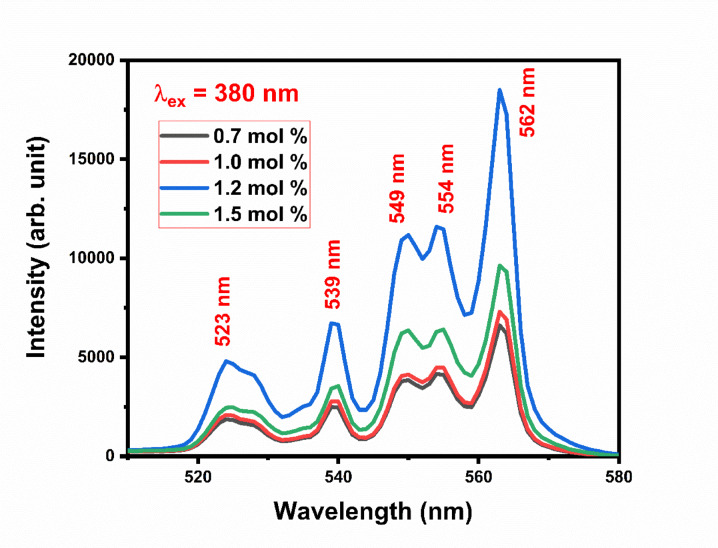
The emission spectra of Gd_2_O_3_:Er^3+^ with excitation wavelength of 380 nm.

**Fig. 21:**
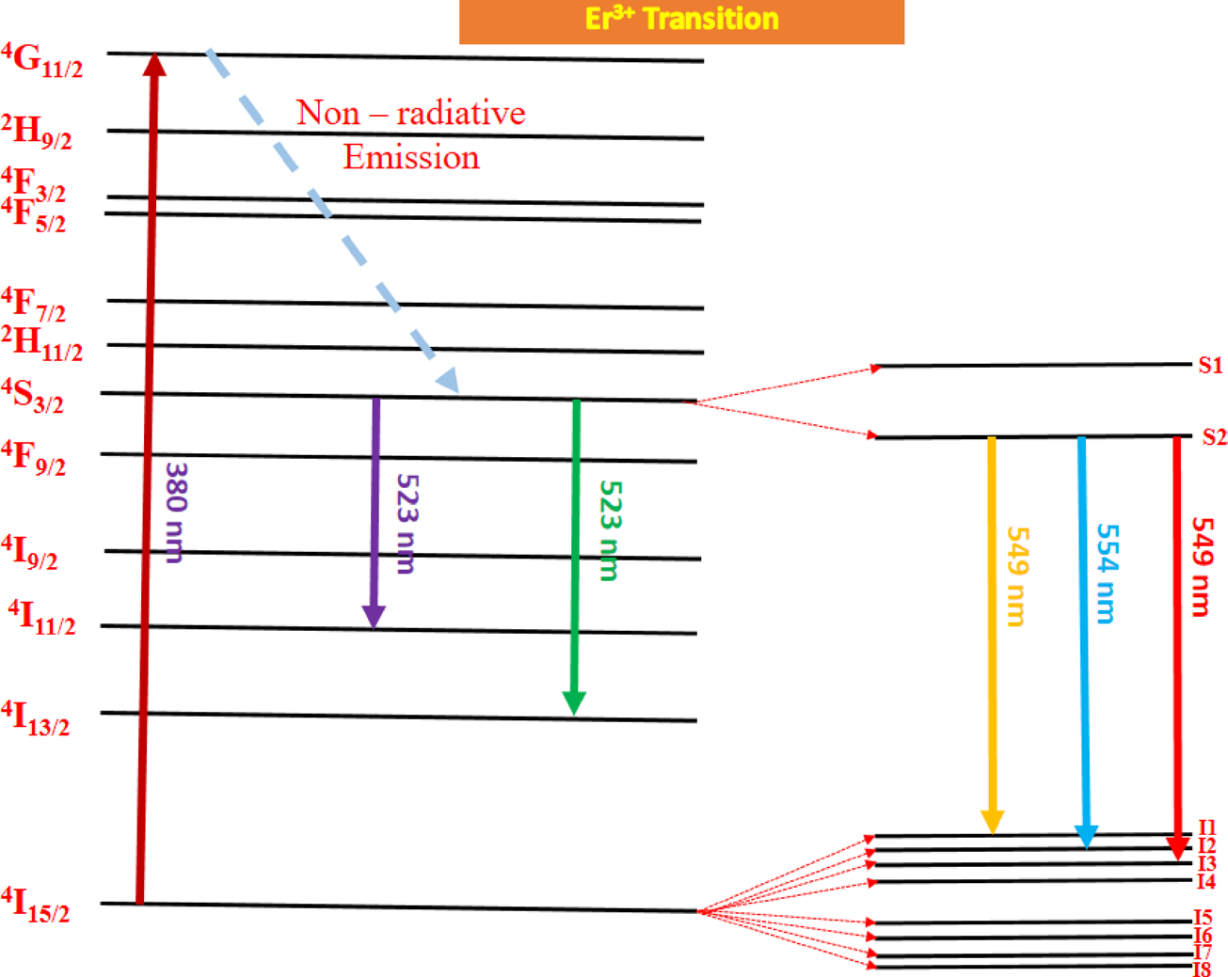
Energy level diagram of Er^3+^.

**Fig. 22:**
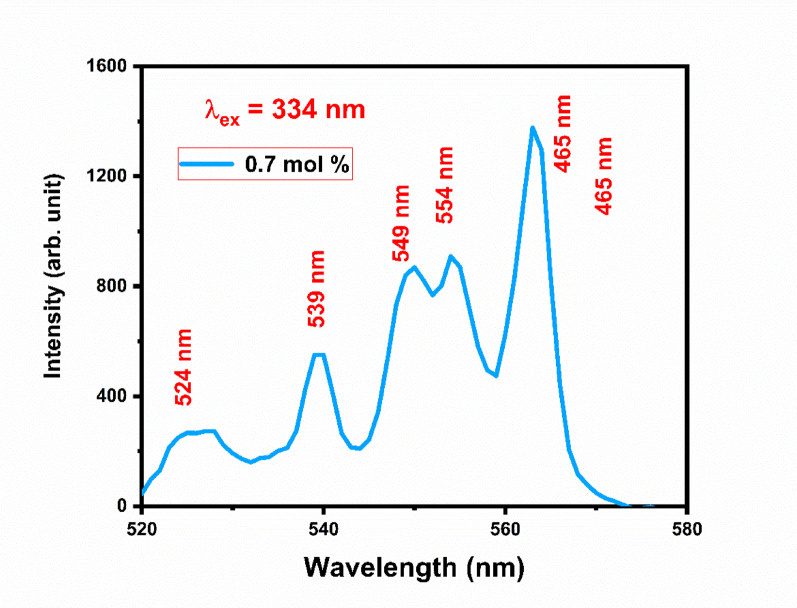
Emission spectra of Gd_2_O_3_:Er^3+^(0.7 mol %) at excitation wavelength 334 nm.

**Fig. 23:**
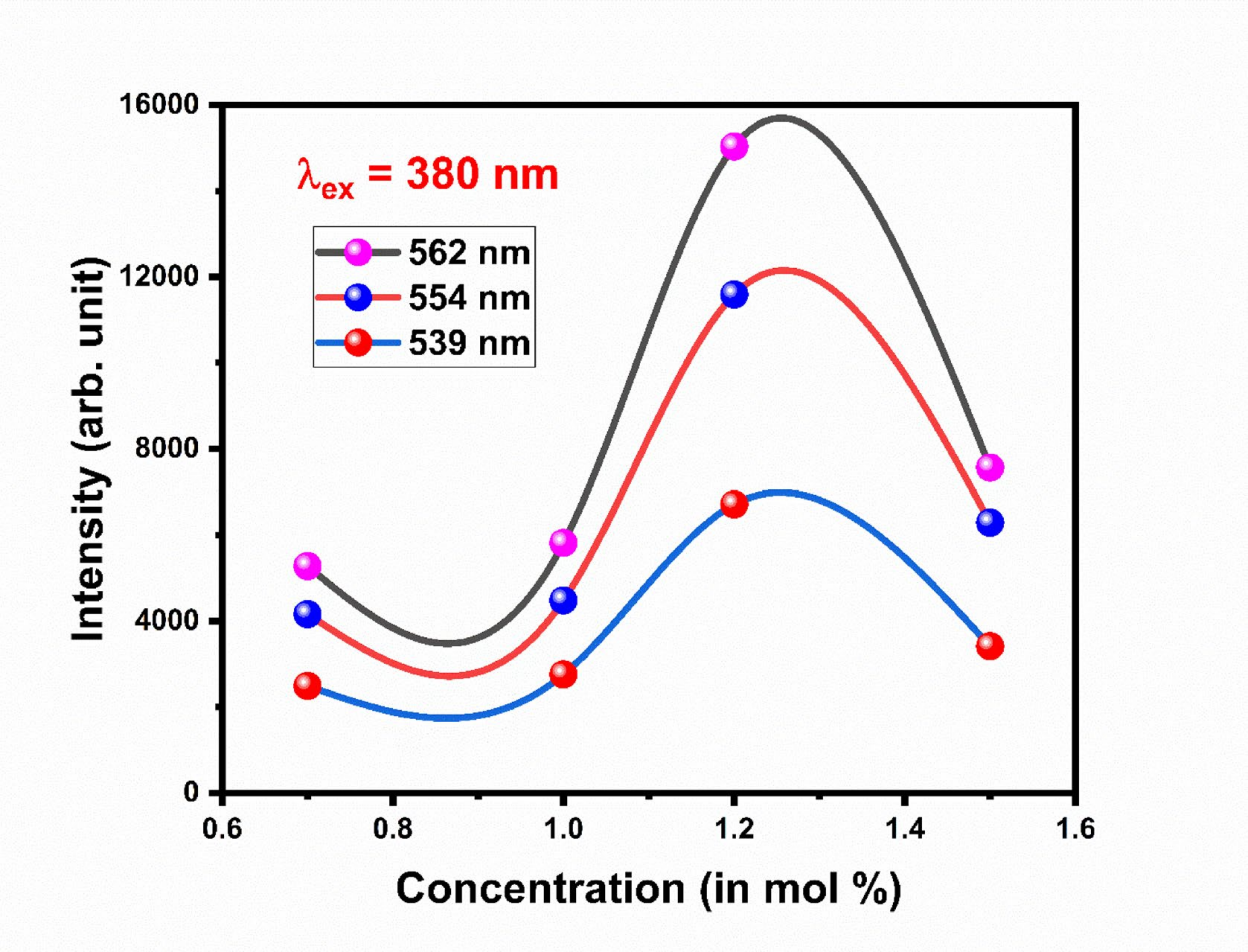
Concentration Quenching of Gd_2_O_3_:Er^3+^.

Figure 20 shows excitation spectra of Gd_2_O_3_:xEr^3+^, where x = 0.7, 1.0, 1.2, and 1.5 mol % at excitation wavelength 380 nm. The emission spectra have 5 peaks centered at 523 nm, 539 nm, 549 nm, 554 nm, and 562 nm. Peaks at 523 nm, and 539 nm are due ^4^S_2/2_ → ^4^I_11/2_, and ^4^S_2/2_→ ^4^I_13/2_ transition respectively. ^4^S_2/2_ → ^4^I_13/2_ transition responsible for peaks at 549 nm, 554 nm, and 562 nm. Three different peaks of emission due to stark level splitting in crystal structure. Splitting happens mainly due to non-centrosymmetric site environment for dopant ion (Er) and lattice distortion in terms of lattice contraction. While the I shell of the Er ion will break into eight subshells, the S shell of the Er ion will split into two sublevels. So there are numbers of transition are possible. Peak at 549 nm, 554 nm, and 562 nm is equal to 18,215 cm^−1^, 18,051 cm^−1^, and 17,794 cm^−1^, respectively. By comparing the difference between the transition we can conclude that the possible transition of peak at is 549, 554, and 562 nm ^4^S_2/2_ (S2) → ^4^I_15/2_(I1), ^4^S_2/2_ (S2) → ^4^I_15/2_(I2), and ^4^S_2/2_ (S2) → ^4^I_15/2_(I3), respectively as shown in the Fig. 21. Gd_2_O_3_:Er^3+^ (0.7 mol %) phosphor is trigged by excitation wavelength 334 nm. The behavior of emission spectra is the same as compared above with less intensity (Fig. 22). As we increase the concentration of Er ion the intensity increases until the concentration of Er at 1.2 mol % after that the intensity starts to decrease This phenomenon is due to concentrating quenching this may happen due to the agglomeration of dopant ion in the host lattice. Figure 23 shows the concentration quenching of Gd_2_O_3_:Er^3+^ of wavelength 562 nm, 554 nm, and 539 nm^30–32^.

Variation of intensity of the prepared sample when the energy level is impacted by the interaction between ion and that strength is found by using the equation below. Relation of emission intensity and activator ion concentration can be estimated by using below equation^37^.

1 $${{\frac{I}{ x}=k[1+ \beta .x}^{\frac{\theta }{3}} ]}^{-1}$$

Above equation also modified as :

2 $$\mathit{log}\frac{I}{x}=c-\frac{\theta }{3}\mathit{log}x$$

**Fig. 24:**
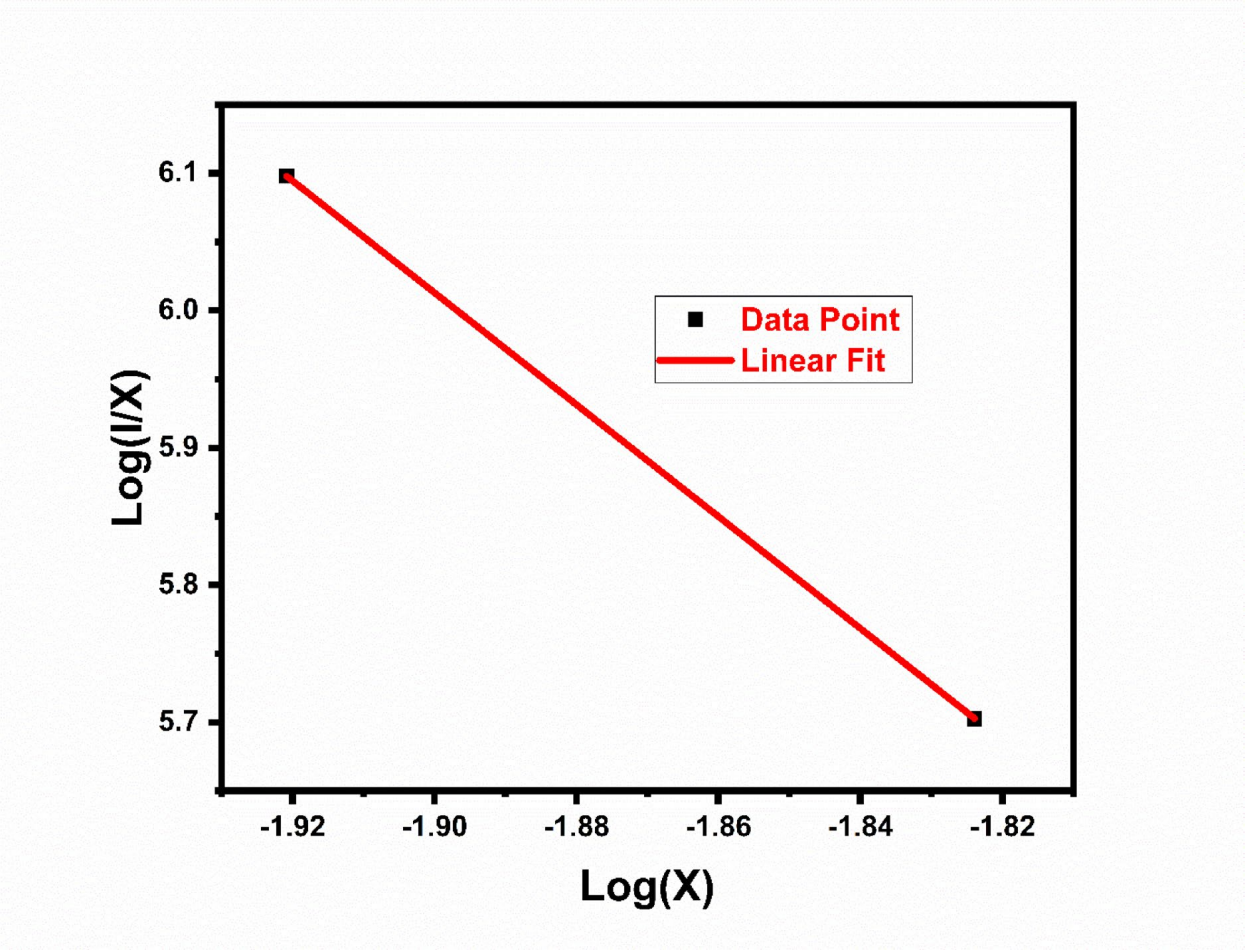
Log(I/X) vs Log(X) of Gd_2_O_3_:Er^3+^

In above equation k and β are constants having identical matrix and same excitation condition. Figure 24 contain graph which gives the relationship between Log(I/x) and Log(x). Slope of Log(I/x) vs log(x) of La_2_O_3_:Eu is 3.278 Slope factor of is found to be 9.834. The value of slope factor is near about 10 so quadrupole–quadrupole interaction is confirmed^38^.

**Fig. 25:**
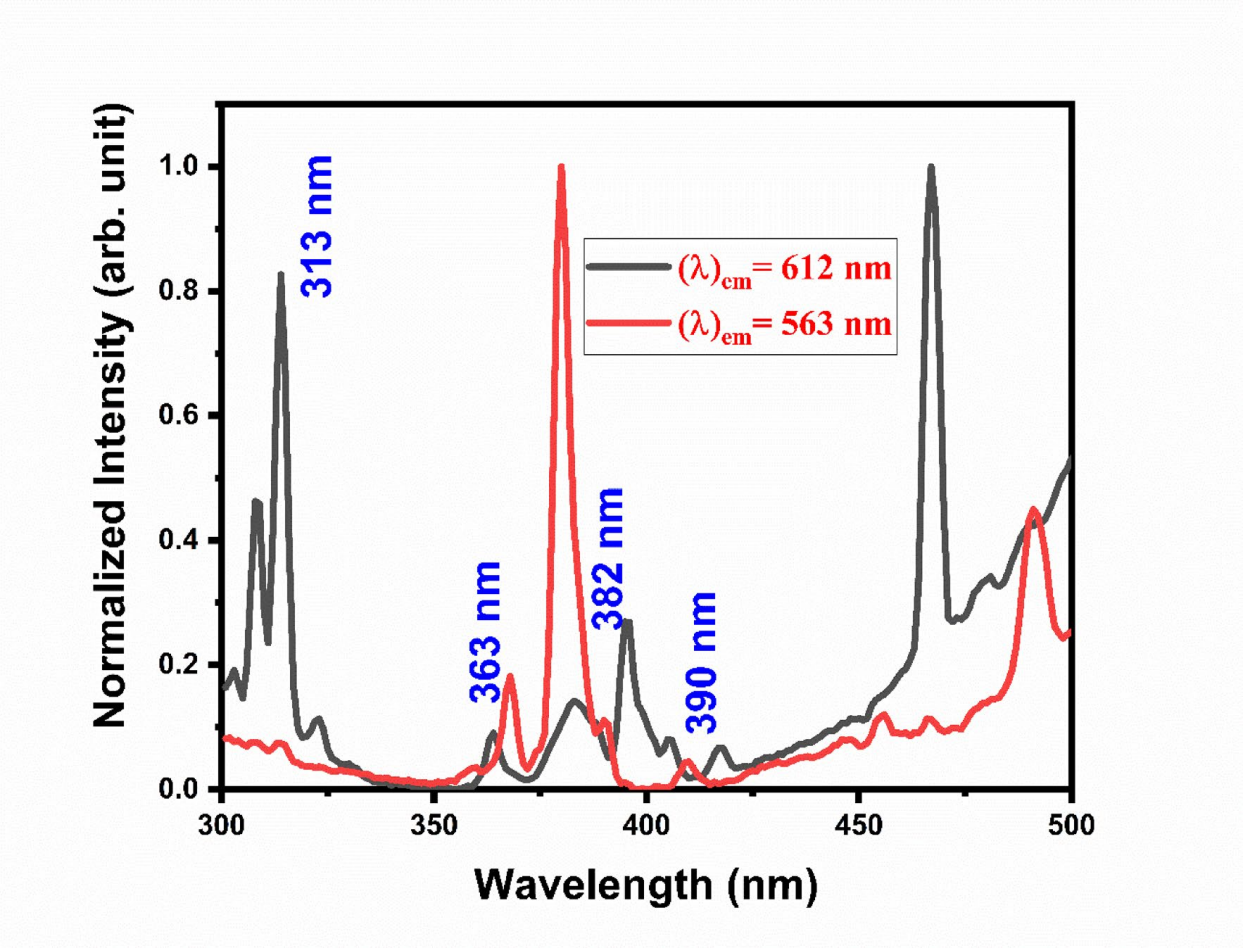
Excitation-Excitation spectra overlap of Gd_2_O_3_:Er^3+^/Eu^3+^.

Figure 25 shows the overlap spectra Gd_2_O_3_:Er^3+^ (1.2 mol %)/Eu^3+^(2.0 mol %). In Fig. 25 the black line graph shows an excitation spectra at emission wavelength 612 nm which corresponds to the Eu transition, while the red graph shows excitation spectra at emission spectra 563 nm which corresponds to the Er transition. In excitation spectra, the peak is due to the transition of host lattice Gd at 313 nm. Peak at 313 is more prominent than 307 nm indicating the co-doped phosphor dopant is substituted at some specific site in the host lattice. In the above overlap spectra, both spectra overlap at 363 nm, 382 nm, and 390 nm. In excitation-excitation overlap energy is transferred from a lower wavelength to a higher wavelength, in excitation-emission overlap energy is transferred from an emission ion wavelength to an excitation ion wavelength, and in emission-emission overlap energy is transferred from a higher wavelength to a lower wavelength. In our study, we have found the excitation-excitation overlap therefore energy will transfer from a lower wavelength (Er) to a higher wavelength (Eu). To study the emission spectra of Gd_2_O_3_:Er^3+^/Eu^3+^, we kept the Er constant at 1.2 mol% (quenching concentration) and kept varying the concentration of Eu ion.

**Fig. 26:**
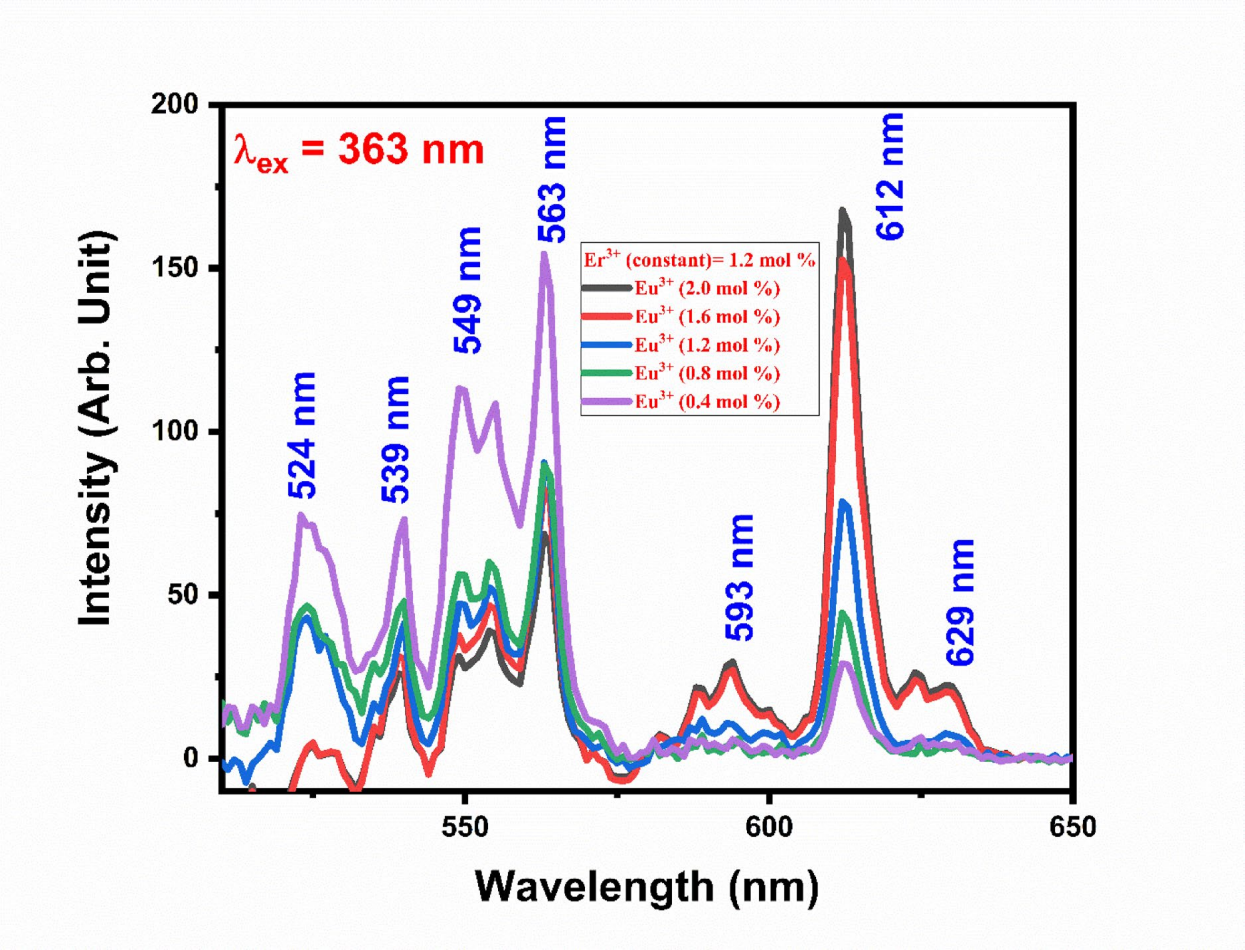
Emission spectra of Gd_2_O_3_:Er^3+^/Eu^3+^ at excitation wavelength 363 nm.

**Fig. 27:**
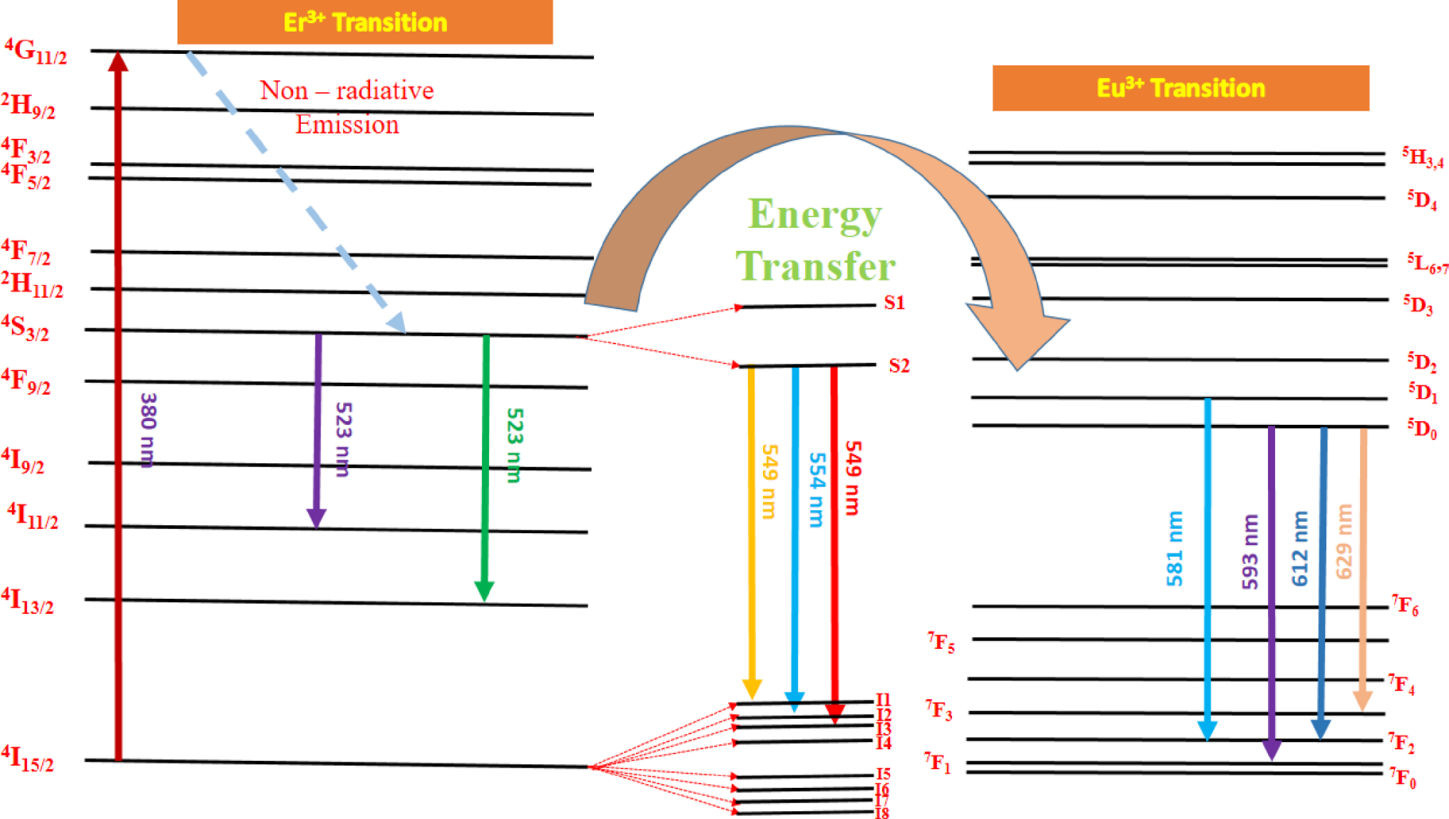
Energy level diagram of Gd_2_O_3_:Er^3+^/Eu^3+^.

**Fig. 28:**
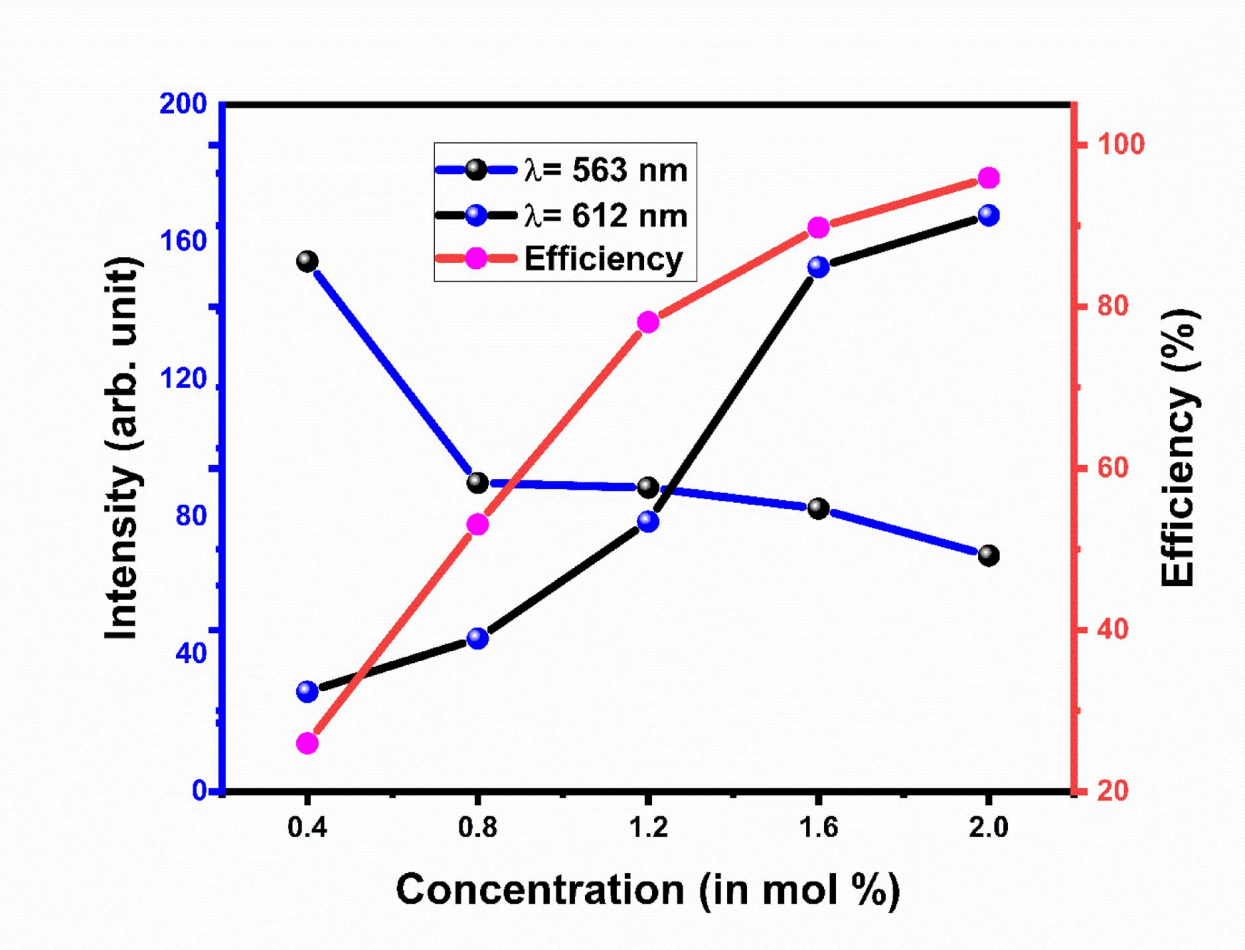
Concentration quenching Gd_2_O_3_:Er^3+^/Eu^3+^ and Energy transfer efficiency of Gd_2_O_3_:Er^3+^/Eu^3+^.

Emission spectra Gd_2_O_3_:Er^3+^(1.2 mol%)/Eu^3+^(0.4–2.0 mol %) shown in Fig. 26. Emission spectra contain 7 peaks 524 nm, 539 nm, 549 nm, 563 nm, 593 nm, 612 nm, and 629 nm. Out of 7 peaks, peaks at 524 nm, 539 nm, 549 nm, 563 nm, and 593 nm are due to the transition of Er respectively. The peak at 593 nm, 612 nm, and 629 nm is due to the transition of Eu respectively. As the concentration of Eu increases, the intensity of the peak due to the transition of Er starts to decrease and the intensity of Eu increases continuously as shown in the Fig. 26. Energy transfer mechanism is shown in Fig. 27. Figure 28 shows varying intensities of peak 563 nm and 612 nm. The efficiency of energy transfer is calculated by using following equation:

3 $$\eta_{T} = 1 - \frac{{I_{Er} }}{{I_{Ero} }}*100 \%$$

where I_Er_ and I_Ero_ are intensity peaks with Er^3+^ and without Eu^3+^ respectively. The energy transfer efficiency of Gd_2_O_3_:Er^3+^/Eu^3+^ is calculated as function of concentration of Er^3+^ and shown in Fig. 28. The value of efficiency varies from 25.98 to 95.93% with maximum energy transfer at Gd_2_O_3_:Er^3+^ (1.2 mol%)/Eu^3+^(2.0 mol %). Detailed energy transfer is shown in the Table 5.

**Table 5:** Energy transfer efficiency of Gd_2_O_3_:Er^3+^/Eu^3+^.

sr	Name	I_Er_	I_Ero_	Efficiency (%)
1	Gd_2_O_3_:Er^3+^(1.2 mol%)/Eu^3+^(0.4 mol%)	167.765	226.651	25.98091
2	Gd_2_O_3_:Er^3+^(1.2 mol%)/Eu^3+^(0.8 mol%)	152.6662	325.214	53.05671
3	Gd_2_O_3_:Er^3+^(1.2 mol%)/Eu^3+^(1.2 mol%)	78.587	358.504	78.07918
4	Gd_2_O_3_:Er^3+^(1.2 mol%)/Eu^3+^(1.6 mol%)	44.547	436.275	89.78924
5	Gd_2_O_3_:Er^3+^(1.2 mol%)/Eu^3+^(2.0 mol%)	29.081	714.829	95.93175

### CIE chromaticity

Luminescent properties of Gd_2_O_3_:Eu^3+^, Gd_2_O_3_:Er^3+^ and Gd_2_O_3_: Er^3+^ / Eu^3+^ were calculated by using the Commission de l’Eclairage (CIE) coordinates. CIE coordinates provide information about the color of the emission spectra. With the help of CIE co-ordinate, we can find color purity and correlated color temperature (CCT). To find the color purity

4 $$Color\; purity=\frac{\sqrt{{\left(X-{X}_{i}\right)}^{2}+{\left(Y-{Y}_{i}\right)}^{2}}}{\sqrt{{\left({X}_{d}-{X}_{i}\right)}^{2}+{\left({Y}_{d}-{Y}_{i}\right)}^{2}}}*100 \%$$

Here (X,Y) is CIE Chromaticity coordinate obtained CIE daigram, while (X_i_,Y_i_)are CIE co-ordinate of perfect white light and (X_d_, Y_d_) are CIE co-ordinate of dominant wavelength in emission spectra^39^. The correlated color temperature (CCT) of the phosphor can be obtained by below using equation:

5 $${\text{CCT}} = - {\text{ 437n}}^{{3}} + { 36}0{\text{1n}}^{{2}} + - {\text{ 6861n }} + { 5514}.{31}$$

**Fig. 29:**
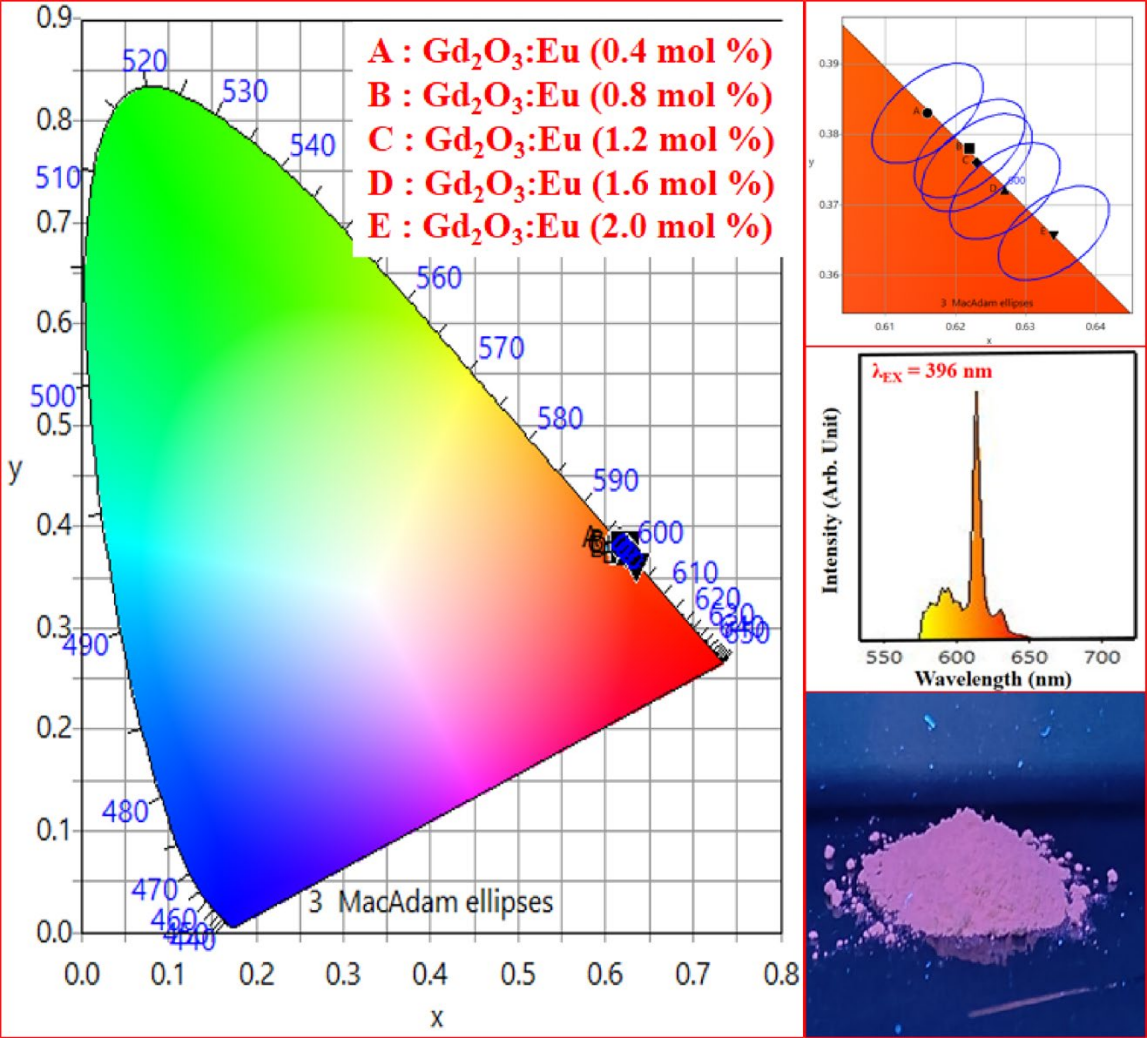
CIE Co-ordinate of Gd_2_O_3_: Eu^3+^and phosphor under UV lamp.

**Fig. 30:**
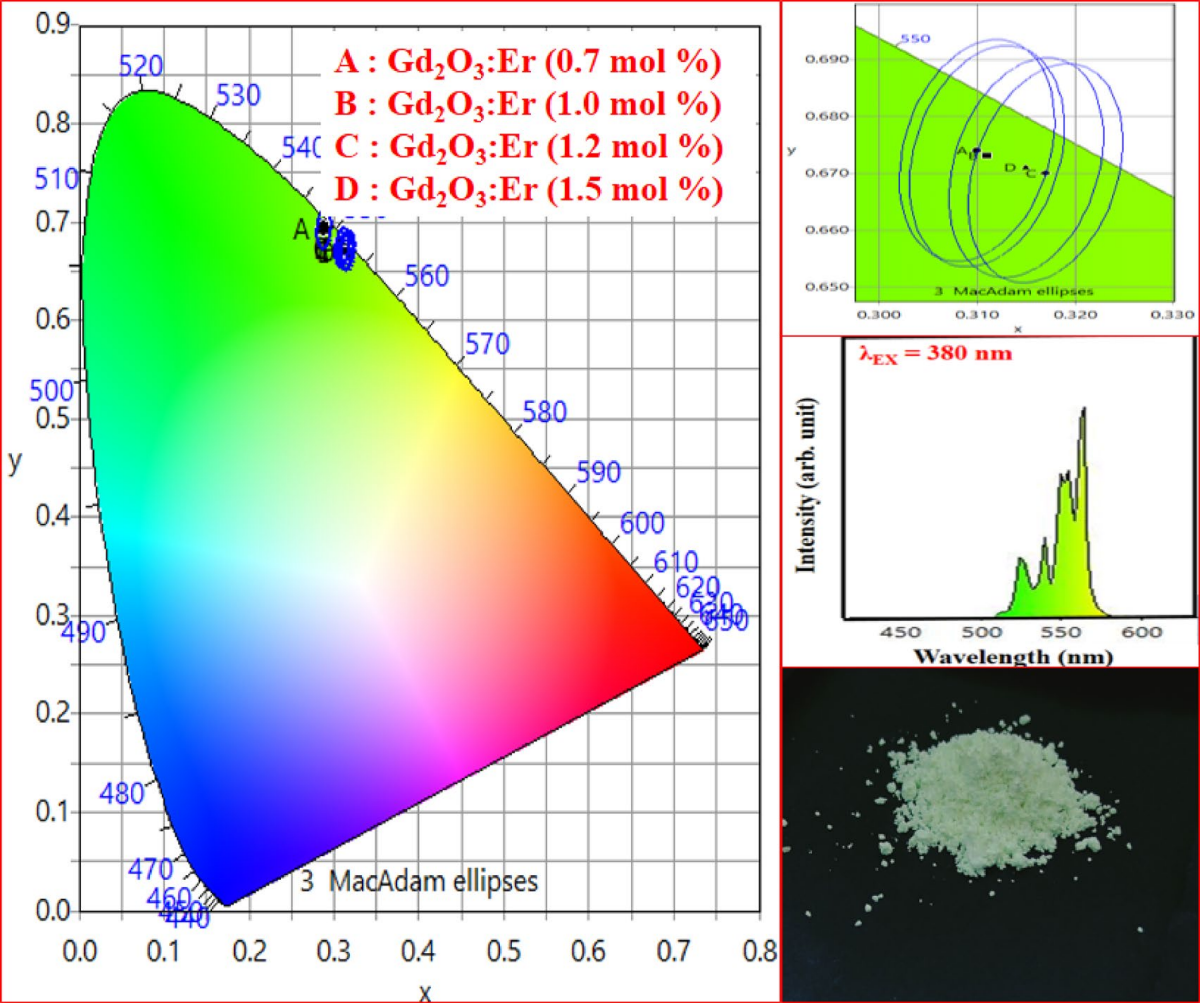
CIE Co-ordinate of Gd_2_O_3_:Er^3+^ and phosphor under UV lamp.

Figure 29 shows the CIE co-ordinate of Gd_2_O_3_:Eu^3+^ at different concentrations of dopant. The emission of phosphor triggered 396 nm falls in the red region. Table 6 contains the CIE coordinate, CCT, and color purity of Gd_2_O_3_:Eu^3+^. As we increases concentration of Eu ions in prepared phosphor, the x-coordinate slightly increases and the y-coordinate slightly decreases, resultant emission shift toward intense red region. Figure 30 shows the image of phosphor under UV lamp. Red emitting phosphor is a prominent candidate for fingerprint detection. These phosphors are also useful in red LEDs and anti-counterfeiting applications. Color purity of the sample ranges from 83.97 to 89.29% with the highest at Gd_2_O_3_:Eu^3+^ (2.0 mol%). This high color purity makes it a prominent candidate for display application. CCT of phosphor ranges from 1796.76 to 2079.18 K. These low CCT phosphors can be used in plant lighting and ambient lighting. Gd_2_O_3_:Eu^3^⁺ shows dominant red emission, which enhances warm color tones but limits CRI due to poor spectral balance^40^.

**Table 6:** Color purity, CIE co-ordinate and CCT of Gd_2_O_3_: Eu^3+^

Gd_2_O_3_: Eu^3+^							
Sr	Name	X	Y	X_d_	Y_d_	CCT (K)	Colour purity (%)
1	Gd_2_O_3_: Eu^3+^(0.4 mol%)	0.616	0.383	0.675	0.325	1796.76	83.97
2	Gd_2_O_3_: Eu^3+^(0.8 mol%)	0.622	0.378	0.675	0.325	1859.95	85.47
3	Gd_2_O_3_: Eu^3+^(1.2 mol%)	0.623	0.376	0.675	0.325	1881.86	85.67
4	Gd_2_O_3_: Eu^3+^(1.6 mol%)	0.627	0.372	0.675	0.325	1946.47	86.67
5	Gd_2_O_3_: Eu^3+^(2.0 mol%)	0.634	0.366	0.672	0.672	2079.18	89.29

Figure 30 shows the CIE coordinate of Gd_2_O_3_:Er^3+^ at excitation wavelength 380 nm. CIE coordinates confirm the emission in the intense green region, as shown in Table 7. As the concentration of Er ion, X-coordinate, and Y-coordinate start to increase and decrease respectively. Phosphor has color purity which makes it a prominent candidate for display application. CCT of prepared phosphor ranges from 5831 to 5765 K. CCT of Gd_2_O_3_:Er^3+^ (1.5 mol%) phosphor is 5765 K is perfect for LED bulbs, smart lighting, and human-centric lighting systems. It also can be used in cool-white light for medical and commercial lighting applications. Gd₂O₃:Er^3^⁺ exhibits strong green emission, giving cooler chromaticity but again with restricted CRI^40^.

**Table 7:** Color purity, CIE co-ordinate and CCT of Gd_2_O_3_: Er^3+.^

Gd_2_O_3_:Er^3+^							
Sr no	Name	X	Y	X_d_	Y_d_	CCT (K)	Colour purity (%)
1	Gd_2_O_3_:Er^3+^(0.7 mol%)	0.310	0.674	0.395	0.604	5831.13	100
2	Gd_2_O_3_:Er^3+^(1.0 mol%)	0.311	0.673	0.395	0.604	5825.32	100
3	Gd_2_O_3_:Er^3+^(1.2 mol%)	0.317	0.670	0.387	0.611	5734.86	100
4	Gd_2_O_3_:Er^3+^(1.5 mol%)	0.315	0.671	0.395	0.597	5765.05	100

**Fig. 31:**
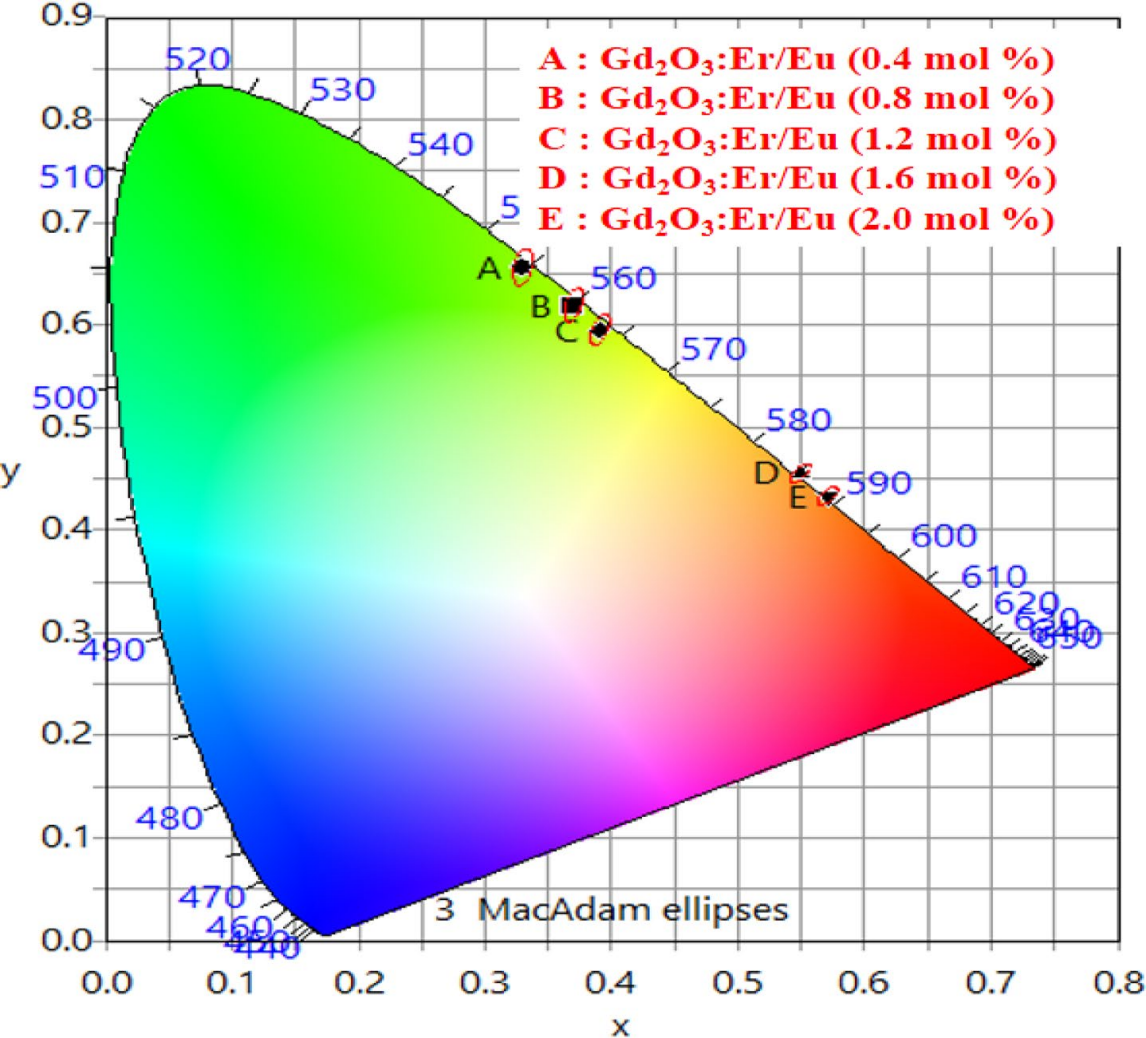
CIE Co-ordinate of Gd_2_O_3_:Er^3+^ /Eu^3+^

**Fig. 32:**
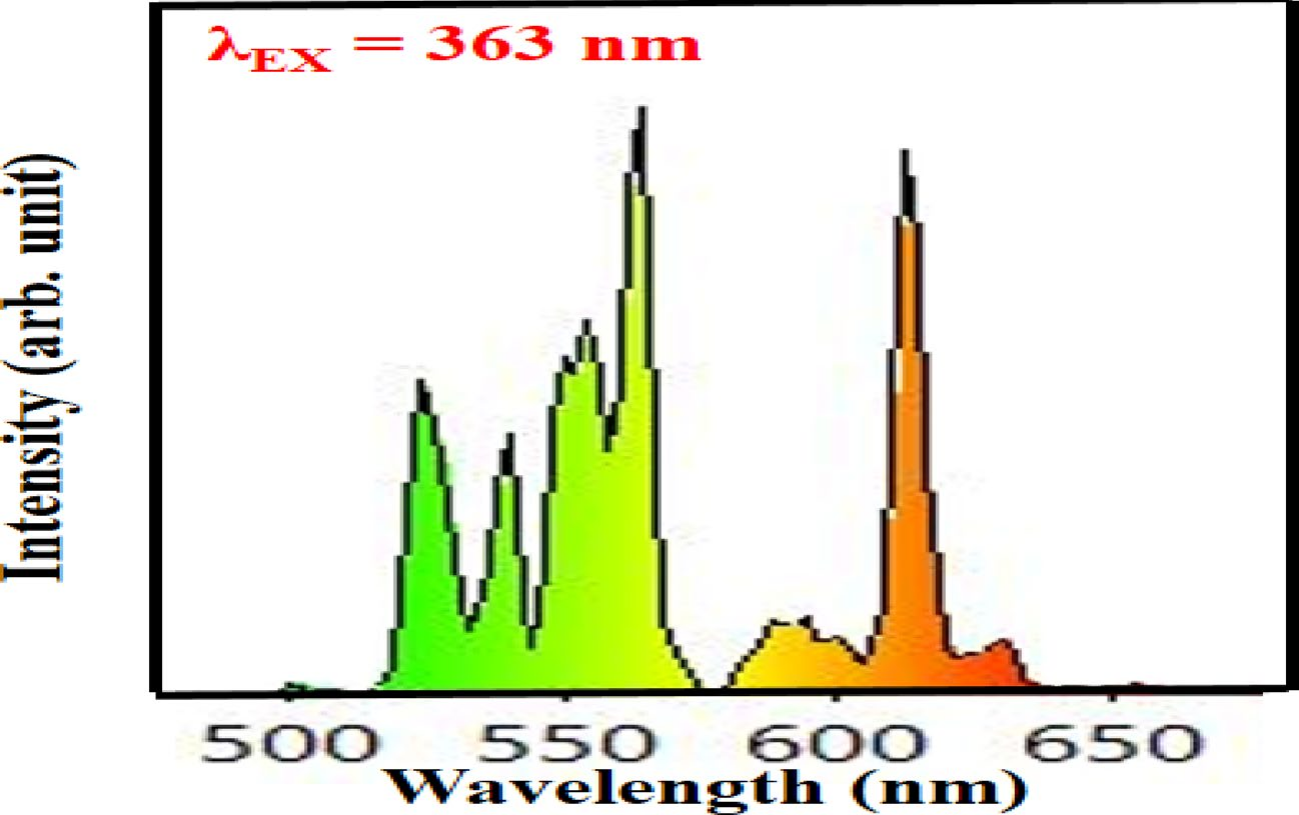
Emission spectra of Gd_2_O_3_:Er^3+^(1.2 mol%) /Eu^3+^(1.2 mol%).

Figure 31 presents the CIE chromaticity diagram of Gd₂O₃:Er^3+^/Eu^3+^ phosphors with varying Eu^3+^ concentrations. As the concentration of Eu^3+^ increases, the CIE coordinates shift progressively from the green region toward the red region, indicating tunable color emission. This color-tunable phosphor can be effectively applied in barcode systems, where a specific composition can be encoded, and under UV illumination, the unique color emission allows for easy detection and identification. However, the color purity of the phosphor decreases from 98.20 to 76.09% with increasing Eu^3+^ concentration. Additionally, the correlated color temperature (CCT) decreases from 5553 to 1856 K, suggesting a transition from cool white to warm/red emission. Detailed numerical data are provided in the accompanying Table 8. Emission spectra of Gd_2_O_3_:Er^3+^(1.2 mol%) /Eu^3+^(1.2 mol%) shown in Fig. 32. Emission spectra of Gd_2_O_3_:Er^3+^(1.2 mol%) /Eu^3+^(0.4 mol%), Gd_2_O_3_:Er^3+^(1.2 mol%) /Eu^3+^(0.8 mol%), Gd_2_O_3_:Er^3+^(1.2 mol%) /Eu^3+^(1.6 mol%) and Gd_2_O_3_:Er^3+^(1.2 mol%) /Eu^3+^(2.0 mol%) shown in supplementary data S3, S4, S5 and S6. Gd_2_O_2_:Eu^3+^, Er^3+^ co-doped phosphor combines red and green emissions, enabling better color conversion and higher CRI through improved chromatic blending^40^.

**Table 8:** Color purity, CIE co-ordinate and CCT of Gd_2_O_3_: Er^3+^/Eu^3+^.

Gd_2_O_3_:Er^3+^/Eu^3+^							
sr	Name	X	Y	X_d_	Y_d_	CCT (K)	Colour purity (%)
1	Gd_2_O_3_:Er^3+^(1.2 mol%)/Eu^3+^(0.4 mol%)	0.330	0.656	0.395	0.604	5553.07	98.2
2	Gd_2_O_3_:Er^3+^(1.2 mol%)/Eu^3+^(0.8 mol%)	0.370	0.619	0.402	0.597	4946.89	96.8
3	Gd_2_O_3_:Er^3+^(1.2 mol%)/Eu^3+^(1.2 mol%)	0.391	0.595	0.395	0.604	4604.36	86.52
4	Gd_2_O_3_:Er^3+^(1.2 mol%)/Eu^3+^(1.6 mol%)	0.549	0.455	0.672	0.328	2096.55	73.11
5	Gd_2_O_3_:Er^3+^(1.2 mol%)/Eu^3+^(2.0 mol%)	0.571	0.433	0.672	0.328	1856.85	76.09

## Strategic applications of Gd_2_O_3_:Er^3+^ /Eu^3+^ phosphor

The Gd_2_O_3_:Er^3+^ /Eu^3+^ phosphor is a color-tunable whose emission can be adjusted by varying the dopant concentration. Nowadays, ensuring the security of important merchandise, critical documents, and currency notes against counterfeiting is a major concern. This phosphor emits light in five different regions, as shown in the CIE diagram.

### Barcode detection

**Fig. 33:**
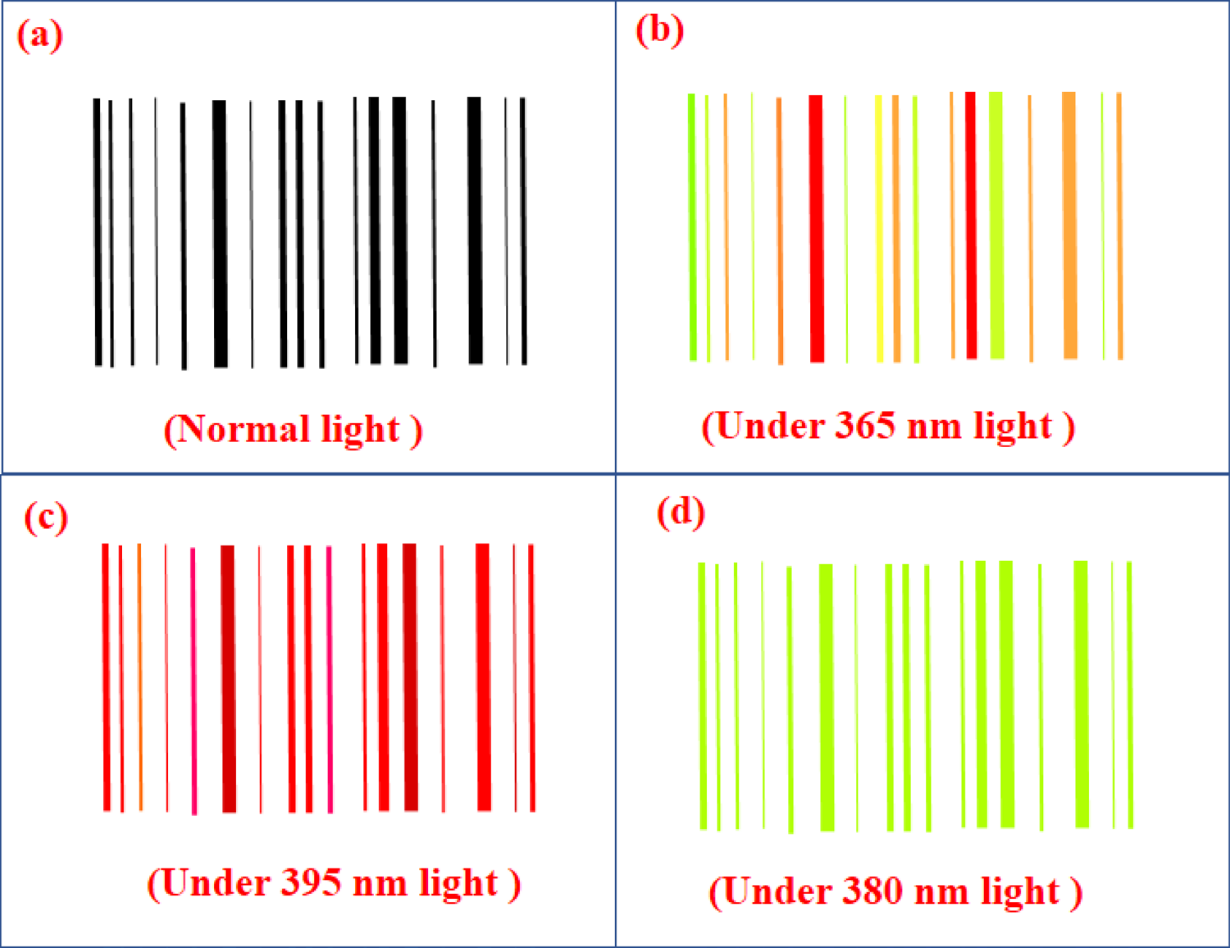
Barcode (**a**) under normal light, (**b**) under 365 nm light, (**c**) under 395 nm light and, (**d**) under 380 nm light.

Barcodes are structured patterns of lines that encode information for scanning and identification. With advancements in technology, replicating traditional barcodes has become increasingly feasible, raising concerns about their use for high-security applications. To address this, we propose a novel approach that incorporates phosphor-based materials into barcodes to enhance security and prevent duplication. This method utilizes a specially designed ink composed of Gd₂O₃:Er^3+^/Eu^3^⁺ phosphors, mixed with a resin and hardener. This phosphor ink is applied over standard barcodes, transforming them into security-enhanced barcodes with unique optical properties when exposed to ultraviolet (UV) light. Figure 33a shows a standard barcode. When coated with the Gd₂O₃:Er^3+^/Eu^3+^ phosphor ink and irradiated with UV light at 365 nm (selected due to its commercial availability and proximity to the phosphor’s excitation peak at 363 nm), the barcode exhibits five distinct emission colors. These emissions correspond to encoded data that can be decoded to verify authenticity, as shown in Fig. 33b**.** To further increase security, we propose filling the spaces between the barcode lines with the phosphor ink. This cloning attempts by complicating the replication of both the emission spectrum and the spatial distribution of the phosphor. Even if an adversary were to attempt duplication using different phosphors that mimic the 365 nm emission colors, additional layers of verification can be employed. For instance:

- Under 395 nm UV light, the barcode displays varied red emission intensities, attributed to different concentrations of Eu^3+^ ions. This variation creates a unique emission pattern that is difficult to replicate precisely, as shown in Fig. 33c.
- Under 380 nm UV light, the barcode consistently emits green light across all bars due to stable Er^3+^ ion emission, providing another layer of authentication as shown in Fig. 33d**.**

This multi-wavelength, multi-emission verification system significantly enhances barcode security, making it suitable for applications requiring robust anti-counterfeiting measures. This feature can also be useful in the anti-counterfeiting of currency notes.

## Conclusion

Photochromic property Gd_2_O_3_:Eu^3+^, Gd_2_O_3_:Er^3+^, and Gd_2_O_3_:Er^3+^/Eu^3+^ were studied. Gd_2_O_3_ doped with Eu^3+^ and co-doped with Er^3+^ phosphors were prepared using the co-precipitation method by using NaOH. XRD study confirms the cubic structure of Gd_2_O_3_ with space group I 21 3. Impurities like Er cause lattice contraction, confirmed by the shift of the XRD pattern toward higher angles, while Eu causes lattice expansion, confirmed by the shift toward smaller angles. Adding both impurities leads to lattice distortion with the dominant Er impurity it confirmed by shifting toward a high angle and reducing intensity. SEM confirms the particles have irregular shapes and sizes, and an average particle size of 401 nm. In Tem analysis of prepared phosphor have some particle are in spherical in shape. FT-IR confirms the Gd-O bond in the host lattice which also confirms the formation of Gd_2_O_3_. Gd_2_O_3_:Eu^3+^ and Gd_2_O_3_:Er^3+^ give the emission in red and green regions, respectively. The energy transfer mechanism of Gd_2_O_3_:Er^3+^/Eu^3+^ is studied with a maximum efficiency of 95.93%. Color-tunable Gd_2_O_2_:Eu^3+^/Er^3+^ phosphors offer enhanced security for barcodes through distinct emission properties. Gd_2_O_2_:Er^3+^ (1.5 mol%) shows strong green emission with a favorable CCT of 5765 K, making it suitable for LED applications. The use of Gd_2_O_2_:Er^3+^/Eu^3+^ phosphor ink adds multiple layers of security to traditional barcodes through unique UV-induced emissions. This innovative approach makes barcode duplication extremely difficult, enhancing their suitability for high-security applications. These multifunctional phosphors hold great promise for both security and lighting technologies.

## Supplementary Information

Below is the link to the electronic supplementary material.

Supplementary Material 1

## Data Availability

The datasets generated and/or analysed during the current study are not publicly available due to intellectual property protection but are available from the corresponding author on reasonable request.
