# Supplementary material for: Tuning photoluminescence in Gd2O3 via lattice engineering for advanced barcode and LEDs applications
Source: Sci Rep. 2025 Oct 28;15:37706. doi: 10.1038/s41598-025-21434-3 (PMC12569252; doi:10.1038/s41598-025-21434-3)
Supplement: Supplementary file 1 — Supplementary Material 1 [file 41598_2025_21434_MOESM1_ESM.docx]

**Supplementary data**

**

**

**Figure S1:** Emission spectra of Gd_2_O_3_:Eu^3+^ at excitation wavelength 314 nm.





**Figure S2:** Emission spectra of Gd_2_O_3_:Eu^3+^ at excitation wavelength 276 nm.

**
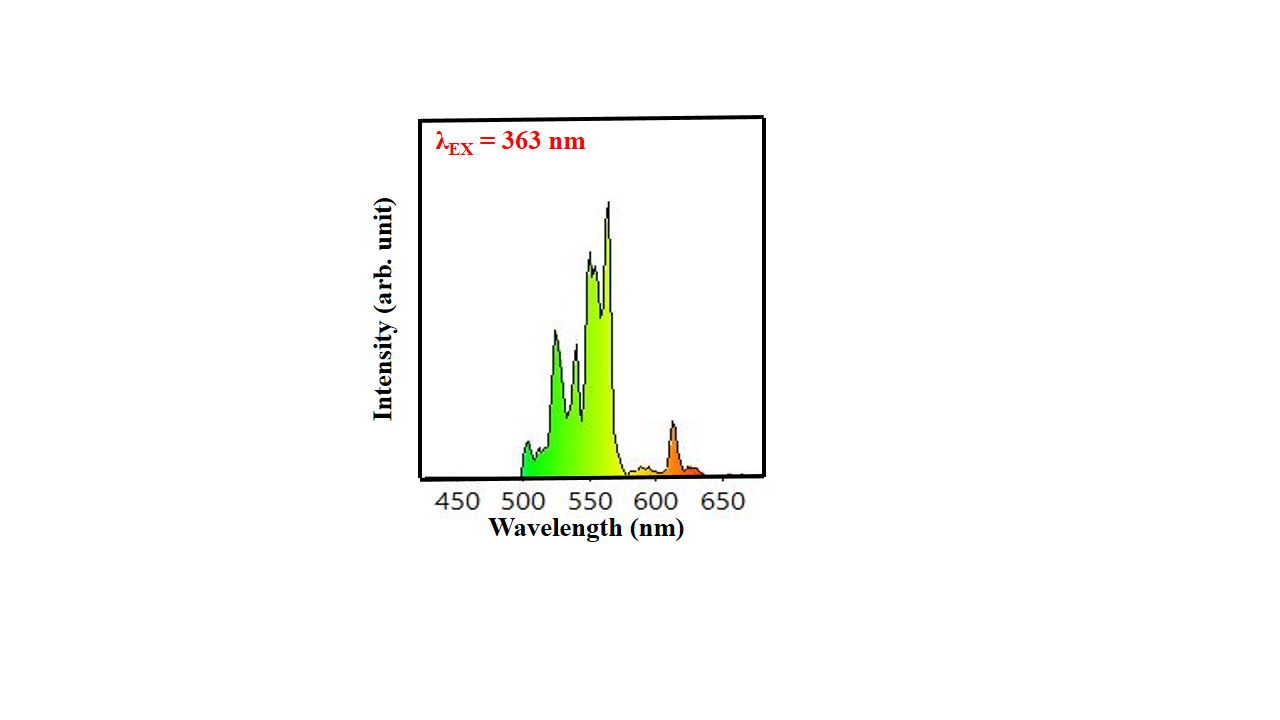
**

**Figure S3**: Emission spectra of Gd_2_O_3_:Er^3+^(1.2 mol%) /Eu^3+^(0.4 mol%)


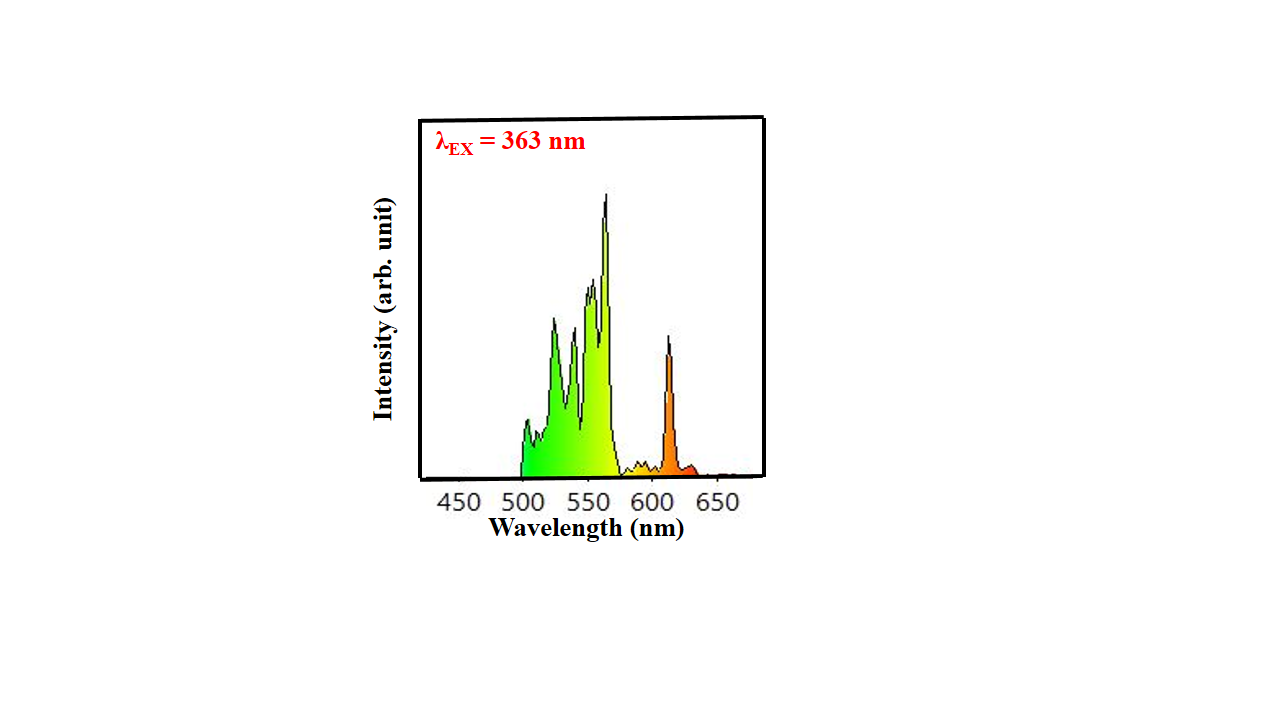


**Figure S4**: Emission spectra of Gd_2_O_3_:Er^3+^(1.2 mol%) /Eu^3+^(0.8 mol%)


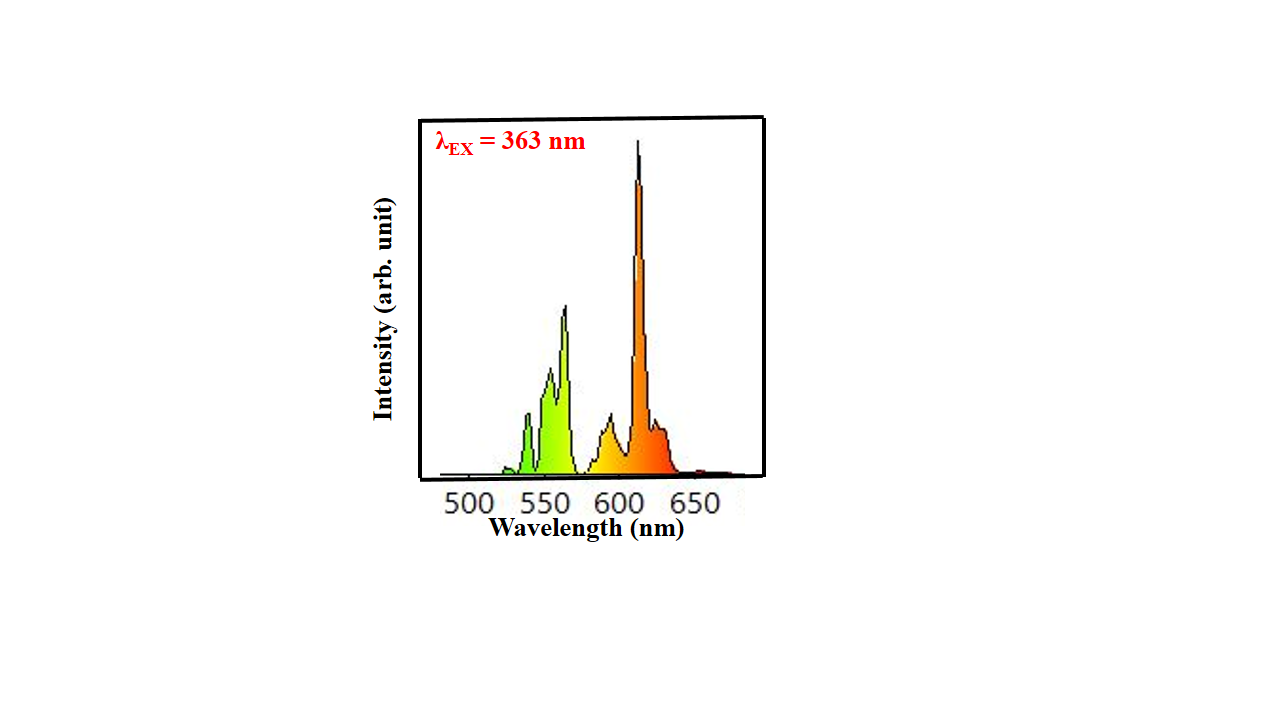


**Figure S5**: Emission spectra of Gd_2_O_3_:Er^3+^(1.2 mol%) /Eu^3+^(1.6 mol%)


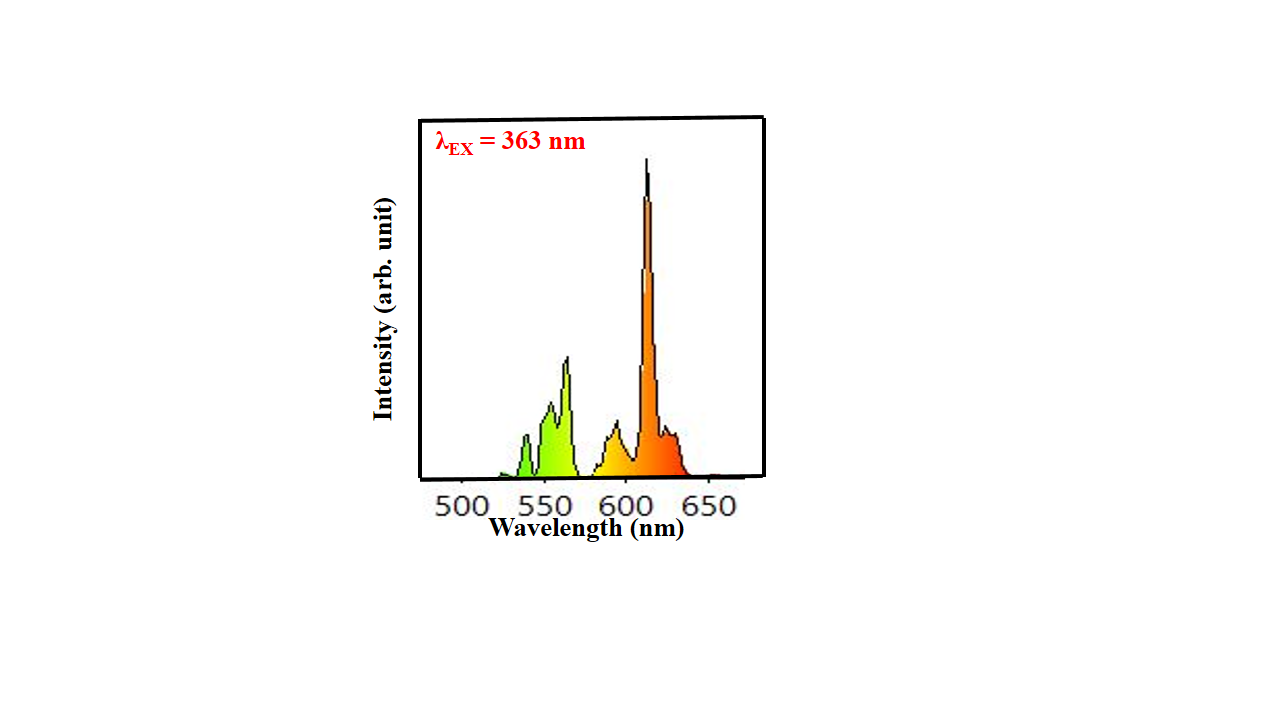


**Figure S6**: Emission spectra of Gd_2_O_3_:Er^3+^(1.2 mol%) /Eu^3+^(2.0mol%)
